# Long Sleep Duration and Multimorbidity in Older U.S. Adults: A Dose–Response Analysis of NHANES 2017–March 2020

**DOI:** 10.3390/healthcare14213580

**Published:** 2026-10-22

**Authors:** Jinhua Zhang, Ki-Hyun Kim, Hyungsoo Shin, Wansuk Choi

**Affiliations:** 1 Department of Medical Technology and Nursing, Laiwu Vocational and Technical College, Jinan 271100, China; lhzjh20181214@gmail.com; 2 Graduate School of Physical Therapy, Kyungwoon University, Gumi 39160, Republic of Korea

**Keywords:** sleep duration, multimorbidity, older adults, NHANES, dose–response, cross-sectional study, effect modification, primary care

## Abstract

**Background/Objectives**: Sleep disturbance and chronic-disease multimorbidity are both common in aging populations, yet the dose–response shape of their association in U.S. older adults, and the sleep threshold of clinical relevance, remain unclear. **Methods**: We analyzed 2998 community-dwelling U.S. adults aged ≥60 years from the NHANES 2017–March 2020 pre-pandemic cycle. Self-reported sleep duration was categorized as short (<6 h), normal (6–8 h), or long (>8 h); multimorbidity was ≥2 of eleven physician-diagnosed chronic conditions. Survey-weighted logistic regression with jackknife variance estimated adjusted odds ratios (aORs), and restricted cubic splines characterized the dose–response shape. **Results**: Multimorbidity prevalence was 64.0%. The excess risk was confined to sleep of ≥9 h (aOR = 1.65, 95% CI 1.22–2.25); the band immediately above the reference, >8 to <9 h, showed no elevation (aOR = 1.08, 0.67–1.73), and the spline was J-shaped (P-nonlinearity = 0.005). Across the conventional >8 h category, long sleep was associated with multimorbidity (aOR = 1.41, 1.08–1.85, *p* = 0.014), whereas short sleep was not (aOR = 0.92, 0.58–1.47). This corresponded to an adjusted risk difference of 7.0 percentage points, and it was not materially attenuated by adjustment for a frailty phenotype (aOR = 1.38, 1.00–1.90) or among participants carrying at most one of the three available frailty criteria (aOR = 1.42, 1.03–1.97). The E-value, corrected for this common outcome, was 1.51. **Conclusions**: Long sleep, particularly ≥9 h, was associated with multimorbidity along a J-shaped dose–response. The association was moderate and the cross-sectional design cannot exclude reverse causation, so long sleep is best interpreted as a marker of accumulated disease burden rather than an actionable risk factor.

## 1. Introduction

The global population aged 65 years and older is projected to reach 2.2 billion by the late 2070s, with the share of older adults nearly doubling globally between 2024 and 2054. This demographic shift has been accompanied by a parallel rise in chronic disease multimorbidity, commonly defined as the co-occurrence of two or more chronic conditions in the same individual [1,2]. In the United States, the prevalence of multimorbidity exceeds 60% among community-dwelling adults aged 65 and over [3], a burden recently quantified in a NHANES-based analysis of US middle-aged and older adults [4], substantially increasing healthcare utilization, functional dependence, polypharmacy, and mortality risk [3,5]. Identifying modifiable behavioral factors associated with multimorbidity in this population is therefore a central priority for geriatric public health.

Sleep is a fundamental component of healthy aging. Both short and long sleep durations have been linked to a wide range of adverse cardiometabolic, neurocognitive, and inflammatory outcomes [6,7,8]. Mechanistically, sleep deviations from optimal duration may dysregulate the hypothalamic–pituitary–adrenal axis, impair glucose tolerance and lipid metabolism, elevate systemic inflammation, and accelerate biological aging [9,10]. In older adults specifically, prolonged sleep has been hypothesized to reflect underlying inflammatory burden, undiagnosed depression, sarcopenia, or subclinical cognitive decline, which may concurrently drive the accumulation of chronic disease [11,12,13]. National Sleep Foundation guidelines recommend 7–8 h of nightly sleep for older adults, yet whether deviations from this range relate to the cumulative burden of multimorbidity, rather than to individual diseases, remains uncertain.

Despite this growing body of evidence, several gaps remain. First, although recent work has begun to link sleep to multimorbidity, the evidence derives largely from longitudinal cohorts outside the United States or from syntheses of heterogeneous studies [14,15], whereas most earlier research addressed individual chronic diseases (e.g., diabetes, cardiovascular disease) rather than their cumulative burden. Second, dose–response analyses in older adults have produced inconsistent findings, with reports of U-shaped, J-shaped, and monotonic relationships [16,17]. Third, subgroup-specific effects (particularly across sex, age strata, adiposity status, and socioeconomic position) are often described informally without formal tests of effect modification, which can lead to overinterpretation of apparent heterogeneity [18]. Fourth, even where nationally representative U.S. data have been analyzed, the dose–response shape of the sleep–multimorbidity relationship has not been characterized with restricted cubic splines under a full complex-survey design, nor has its robustness to unmeasured confounding been quantified (e.g., via the E-value).

To address these gaps, we used the most recent nationally representative pre-pandemic cycle of the National Health and Nutrition Examination Survey (NHANES 2017–March 2020) to: (i) estimate the association between sleep duration categories and multimorbidity in adults aged ≥60; (ii) characterize the dose–response shape using restricted cubic splines; (iii) assess heterogeneity across pre-specified subgroups with formal interaction testing; and (iv) evaluate robustness through multiple sensitivity analyses including alternative outcome definitions and unmeasured-confounding bounds. We hypothesized that long sleep duration would be independently and non-linearly associated with multimorbidity, and that the magnitude of this association would be broadly consistent across major subgroups. Clarifying whether excess risk emerges only at the extreme of the long-sleep range (e.g., ≥9 h) or across the entire >8 h spectrum may inform how sleep duration is interpreted during routine geriatric assessment.

## 2. Materials and Methods

### 2.1. Study Design and Data Source

We conducted a cross-sectional analysis using publicly available data from the National Health and Nutrition Examination Survey (NHANES) 2017–March 2020 pre-pandemic cycle. Administered by the National Center for Health Statistics (NCHS) of the U.S. Centers for Disease Control and Prevention, NHANES draws a stratified, multistage, clustered probability sample and applies sampling weights so that the resulting estimates reflect the non-institutionalized U.S. civilian population [19]. The 2017–March 2020 cycle combines data from the 2017–2018 and 2019–March 2020 cycles, the latter of which was prematurely terminated due to the COVID-19 pandemic; NCHS subsequently released these combined files with revised sample weights to ensure national representativeness [20]. All NHANES protocols were approved by the NCHS Research Ethics Review Board, and written informed consent was obtained from all participants. The present analysis used de-identified public-use data and was therefore exempt from additional institutional review board approval. We followed the Strengthening the Reporting of Observational Studies in Epidemiology (STROBE) guidelines for cross-sectional studies [21].

### 2.2. Study Population

**Figure 1:**
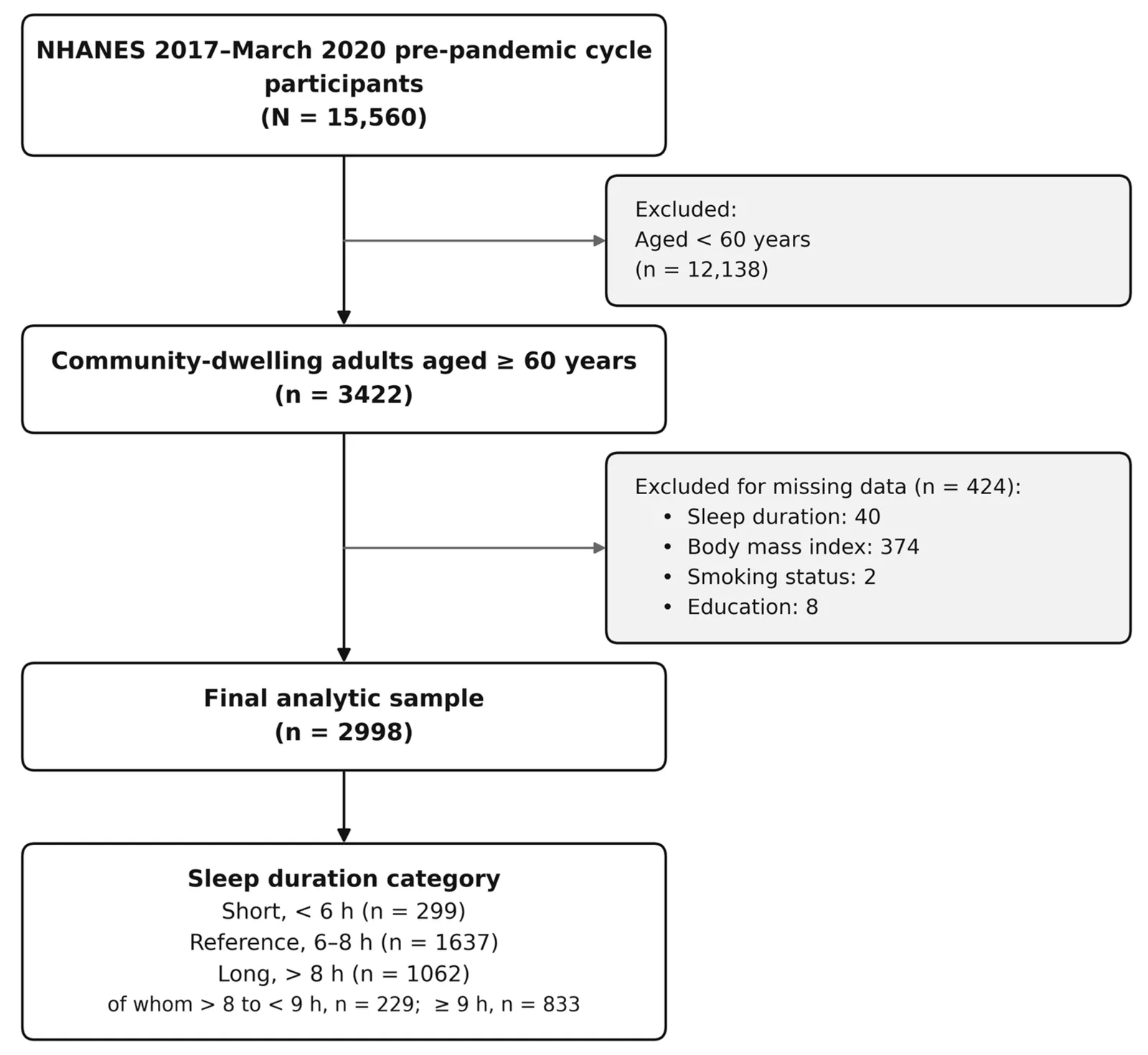
Participant flow diagram. Derivation of the analytic sample from the NHANES 2017–March 2020 pre-pandemic cycle. Exclusions for missing data were applied sequentially in the order shown, so a participant already excluded at an earlier step is not counted again at a later one. NHANES, National Health and Nutrition Examination Survey.

Of 15,560 participants in the 2017–March 2020 cycle, we restricted the analytic sample to community-dwelling adults aged ≥60 years (*n* = 3422). After excluding individuals with missing data on sleep duration (*n* = 40), multimorbidity ascertainment, body mass index, smoking status, or sociodemographic covariates, the final analytic sample comprised 2998 participants (Figure 1). The complex design reduces the information available for inference (Kish effective sample size 1213; design effect 2.47 from unequal weighting), and the short-sleep stratum is small (weighted 8.0%, *n* = 299), so estimates within that stratum are correspondingly imprecise. Included and excluded participants are compared, and the missing-data mechanism is formally assessed, in Section 2.6 and the Supplementary Material. We do not report a post hoc power calculation: for a completed analysis of an existing survey such a calculation is a deterministic function of the observed *p*-value and adds nothing to the confidence intervals reported below.

### 2.3. Exposure Assessment: Sleep Duration

Sleep duration was self-reported during the in-home interview using the Sleep Disorders Questionnaire (variable SLD012), which asks: “How much sleep do you usually get at night on weekdays or workdays?”. Responses were recorded to the nearest half-hour (range 2–14 h); approximately 30% of older participants reported a non-integer value (e.g., 7.5 or 8.5 h), so the distinction between the >8 h and ≥9 h ranges is substantive rather than nominal. Consistent with established sleep epidemiology literature and National Sleep Foundation recommendations for older adults [22], sleep duration was categorized as short sleep (<6 h), normal sleep (6–8 h, the reference category), and long sleep (>8 h). Sleep duration was also retained as a continuous variable for restricted cubic spline (RCS) modeling.

### 2.4. Outcome Assessment: Multimorbidity

We defined multimorbidity as the co-occurrence of two or more (≥2) physician-diagnosed chronic conditions, consistent with the most widely adopted definition in the multimorbidity literature [23,24]. Eleven chronic conditions were ascertained through standardized in-home interviews based on participants’ self-reported affirmative response to “Has a doctor or other health professional ever told you that you had…” each of the following: (1) hypertension (BPQ020); (2) diabetes mellitus, including borderline diabetes (DIQ010); (3) congestive heart failure (MCQ160B); (4) coronary heart disease (MCQ160C); (5) myocardial infarction (MCQ160E); (6) stroke (MCQ160F); (7) arthritis (MCQ160A); (8) chronic obstructive pulmonary disease (MCQ160P); (9) cancer of any type (MCQ220); (10) liver disease (MCQ160L); and (11) weak/failing kidneys (KIQ022).

Three criteria governed this selection. First, every condition had to be ascertained by a standardized physician-diagnosis question of identical form, so that no condition entered the count on a different evidentiary basis from the others. Second, the set had to reproduce the condition lists used in the NHANES multimorbidity literature against which our prevalence estimates are read [25,26]. Third, each condition had to be a chronic, clinically managed diagnosis rather than a symptom, a functional limitation, or a marker of care intensity. These criteria, rather than any judgment about clinical importance, explain the conditions that were left out.

Osteoporosis (OSQ060) meets the first and third criteria and was omitted from the primary count only because it does not appear in the reference lists in [25,26]; we therefore added it in a pre-specified sensitivity analysis. Depression was omitted because this cycle contains no physician-diagnosis item for it: the available instrument is the self-administered PHQ-9, a current-symptom scale, and admitting a score threshold into the count would mix diagnosed disease with present symptom burden in a single sum; because depression is nevertheless central to the sleep literature, we also refitted the model with a PHQ-9 ≥ 10 indicator added as a twelfth and thirteenth condition alongside osteoporosis. Chronic kidney disease entered the count through the KIQ022 self-report of weak or failing kidneys rather than through laboratory staging. NHANES does release serum creatinine and urinary albumin measurements for this cycle, from which eGFR-based stages could in principle be derived, but a laboratory-defined stage is a categorically different ascertainment from a self-reported physician diagnosis, and mixing the two would make the outcome partly a function of measured biochemistry and partly a function of diagnosis and access to care; we therefore kept ascertainment uniform across all eleven conditions. Dementia could not be included at all: the 2017–March 2020 pre-pandemic release contains no dementia-diagnosis item, and the cognitive functioning module NHANES has fielded for adults aged 60 and over in some earlier cycles was not carried into this release, so no dementia indicator could be constructed from the available files.

Congestive heart failure, coronary heart disease, and myocardial infarction were retained as three separate conditions in the primary definition, but they can reflect a single cardiovascular trajectory in the same participant and could therefore inflate the count for cardiovascular patients relative to participants with an equally severe but singly coded condition. Among the 521 participants with at least one cardiovascular diagnosis, 302 carried one, 156 carried two, and 63 carried all three, so the concern is not hypothetical. We therefore repeated the primary analysis with the three diagnoses collapsed into a single cardiovascular disease indicator, giving a nine-condition count.

Responses of “refused” or “don’t know” to a condition item were treated as absence of that condition. This convention can only bias the count downward, so we also repeated the analysis after excluding every participant who gave such a response to any of the eleven items. Secondary outcomes included multimorbidity defined as ≥3 and ≥4 chronic conditions, and the total number of chronic conditions as a continuous outcome. The survey-weighted prevalence of each condition, and the estimate obtained under every alternative outcome definition, are reported in the Results and the Supplementary Material.

### 2.5. Covariates

Based on prior literature and a directed acyclic graph informed by known causal pathways [27], the following covariates were selected a priori as potential confounders and constitute the adjustment set of the primary model: age (continuous, years); sex (male/female); race/ethnicity (non-Hispanic White, non-Hispanic Black, Hispanic, non-Hispanic Asian, other); education (less than high school, high school, some college, college or above); body mass index (BMI; underweight/normal < 25, overweight 25–29.9, obese ≥ 30 kg/m^2^); and smoking status (never, former, current). Race/ethnicity was fitted in exactly the five categories described here and tabulated in Table 1, with the NHANES Mexican American and Other Hispanic groups combined into a single Hispanic category. Only 24 participants (0.42% weighted) were underweight (BMI < 18.5), too few to support a separate stratum; modeling BMI in four categories with underweight separated changed the long-sleep estimate by 0.001 on the odds-ratio scale.

Three variables were used only in sensitivity analyses and never in the primary model: the family poverty income ratio (PIR; continuous), depressive symptoms measured by the Patient Health Questionnaire-9 (PHQ-9; continuous score) and by an 8-item version with the sleep item removed (PHQ-8), and self-reported recreational physical activity.

Sleep quality was characterized with four items from the same Sleep Disorders Questionnaire that provided the exposure: a complaint of trouble sleeping ever reported to a doctor (SLQ050); daytime sleepiness occurring often or almost always (SLQ120 ≥ 3); witnessed snorting, gasping, or pauses in breathing on five or more nights per week (SLQ040 = 3); and habitual snoring on five or more nights per week (SLQ030 = 3). Each item was entered as a covariate individually, and all four were entered together. For the joint-exposure analysis, participants were classified as having poor sleep quality if they reported any of the first three indicators. Snoring was deliberately excluded from that composite: it is reported at almost identical frequency in every sleep-duration stratum (29.9% to 34.4%) and, on its own, indexes an airway characteristic rather than unrefreshing sleep.

Frailty was operationalized primarily as a partial Fried phenotype [28]. Fried’s phenotype comprises five criteria, of which the 2017–March 2020 pre-pandemic release supports three: unintentional weight loss of at least 5% of body weight over the preceding year (derived from WHD020 and WHD050, with intentional loss excluded through WHQ060); absence of any moderate or vigorous recreational physical activity (PAQ650 and PAQ665 both negative); and exhaustion, defined as feeling tired or having little energy on more than half the days (PHQ-9 item DPQ040 ≥ 2). The two remaining criteria, grip strength and gait speed, are unavailable because NCHS did not carry the Physical Functioning file (PFQ) into the pre-pandemic combined release: PFQ is absent from the cycle file listing and a direct request for P_PFQ.xpt returns HTTP 404, while the corresponding two-year 2017–2018 file exists, confirming that the instrument was fielded but not released in combined form. A standard 36- to 49-item frailty index, which is built on activity-of-daily-living and mobility deficits, therefore cannot be constructed in this cycle. The phenotype score (0 to 3 components present) was entered as a covariate, and participants with fewer than 2 of the 3 components were classified, for a restricted analysis, as carrying at most one criterion; under Fried’s convention this group spans robust and pre-frail states, and no Fried category can be assigned when two criteria are unmeasured. None of the three components is a health-care utilization or medication variable, and the score correlates 0.22 with the chronic-condition count.

We additionally constructed an eleven-item deficit-accumulation index (mFI-11) from fair or poor self-rated health, hospitalization in the past year, four or more health-care visits in the past year, a mental-health visit, polypharmacy (≥5 prescriptions), sedative or anxiolytic medication use, PHQ-9 ≥ 10, physical inactivity, underweight, unintentional weight loss, and osteoporosis. This index is reported only as a deliberately over-adjusted sensitivity analysis, and no conclusion is drawn from it. Seven of its eleven items are consequences of having accumulated chronic diagnoses rather than causes of that accumulation: polypharmacy, four or more health-care visits, hospitalization in the past year, a mental-health visit, a sedative or anxiolytic prescription, fair or poor self-rated health, and osteoporosis. Each is strongly associated with the outcome (odds ratios 1.70 to 7.06 after adjustment for the primary covariate set), and the index as a whole correlates 0.47 with the chronic-condition count, more than twice the correlation of the Fried-style phenotype. Conditioning on such variables removes part of the outcome from the model and biases the exposure coefficient toward the null [27]. To separate the two contributions, we also fitted a four-item version (FI-4) with those seven downstream items removed, which is reported as an ordinary sensitivity analysis.

Prescription medication was taken from the prescription medication file (RXQ_RX), restricted to medications reported as currently taken, and summarized as the number of distinct drugs and as a binary indicator of sedative or anxiolytic medication use. We use the term “sedative or anxiolytic medication” rather than “sedative-hypnotic” because the flag does not identify treatment for insomnia: of the 347 flagged prescriptions in the analytic sample, only 18% were hypnotics licensed for insomnia, and the flag is dominated by trazodone, alprazolam, zolpidem, lorazepam, and clonazepam. It therefore indexes exposure to sedating psychoactive medication in general. The count of prescriptions, like the mFI-11, is reported only as an over-adjusted sensitivity analysis, because the number of medications a participant takes is a direct downstream consequence of the number of diagnoses that participant carries.

### 2.6. Statistical Analysis

All estimates were weighted using the Mobile Examination Center weight (WTMECPRP) and were adjusted for the sampling design by declaring the primary sampling units (SDMVPSU) and strata (SDMVSTRA), so that inferences generalize to the U.S. population [29]. Descriptive statistics were presented as survey-weighted means with standard deviations or weighted percentages.

The association between sleep duration categories and multimorbidity was estimated using survey-weighted multivariable logistic regression accounting for the complex survey design (weights, stratification, and PSU clustering). Variances were estimated by the delete-one-PSU jackknife (rescaling the remaining PSUs within each stratum), which is numerically robust to the high condition number of the survey-weighted information matrix; 95% confidence intervals and *p*-values used the t-distribution with degrees of freedom equal to the number of primary sampling units minus the number of strata. For the 2017–March 2020 pre-pandemic combined file the masked variance units comprise 49 pseudo-PSUs nested within 24 pseudo-strata (SDMVSTRA 149 to 172; 23 strata contain 2 pseudo-PSUs and one contains 3) [20]. All 24 strata and all 49 pseudo-PSUs are represented in the analytic sample, so design df = 25 (49 pseudo-PSUs minus 24 pseudo-strata) for every model reported here.

Three nested models were fit: (1) crude (exposure only); (2) adjusted for the demographic and behavioral covariates listed in Section 2.5; and (3) additionally adjusted for PHQ-9 score. Model 2 is the primary model and carries the inference of this paper. Model 3 is reported as a sensitivity analysis only. Because the PHQ-9, the exposure, and the outcome are all recorded at a single visit, depressive symptoms may act as a confounder, a mediator, or a shared consequence of the same underlying process, and presenting Model 3 as the main analysis would presuppose a causal ordering that this design cannot establish.

Dose–response analysis used restricted cubic splines with 4 knots placed at Harrell’s canonical percentiles for a four-knot fit, the 5th, 35th, 65th, and 95th, corresponding to 5.0, 7.0, 8.5, and 10.5 h of sleep [30]. The reference value was set at 7 h. Non-linearity was tested by Wald test on the spline non-linear terms. We report that test under both variance estimators used in this paper, the delete-one-PSU jackknife and Taylor linearization, because the two differ enough here (*p* = 0.005 and *p* = 0.020, respectively) that quoting only the smaller would overstate how firmly non-linearity is established; both remain below 0.05. Confidence bands for the spline were obtained by the delete-one-PSU jackknife method to ensure numerical stability, and the sensitivity of the dose–response shape to the number and placement of knots was examined using 3-, 4-, and 5-knot specifications.

Reference categories differ across analyses by design (6–8 h for the categorical models, 7 h for the spline, and 7–8 h for the 1-h analysis); all are anchored on the 7–8 h range recommended for older adults [22], so each analysis treats this range as the low-risk referent. The 1-h model uses 7–8 h rather than 6–8 h for a specific reason: its purpose is to locate the threshold within the long-sleep range, which requires a referent that is itself a single one-hour band on the same scale as the contrasts being estimated. Retaining the two-hour 6–8 h referent would have absorbed the 6–7 h band into the reference and left the bands above 8 h non-comparable with the bands below 7 h. Sleep was recorded to the nearest half hour, and a reported value of exactly 8.0 h falls within the 7–8 h reference band; the band immediately above the reference is therefore >8 to <9 h, and it is labeled that way throughout rather than as 8–9 h.

Effect modification was assessed across five pre-specified subgroups (sex, age [60–69 vs. ≥70 years], BMI category, smoking status, and PIR strata). Following Gelman and Stern [18], we report stratum-specific aORs alongside formal interaction tests (Wald test on the multiplicative interaction term), recognizing that “one significant, one non-significant” does not constitute statistical evidence of effect modification. Each interaction test is computed on the same categorization of the moderator that is displayed in the corresponding forest plot, so that the test and the strata answer the same question. For BMI, the categorical and continuous tests are not equivalent (*p* = 0.027 categorical, *p* = 0.450 continuous); we report the categorical test, which corresponds to what is shown. Because five subgroups were examined without correction for multiplicity, any single interaction that reaches conventional significance is treated as hypothesis-generating rather than as an established modification of effect.

Sensitivity analyses included: (i) alternative multimorbidity thresholds (≥3 and ≥4 chronic conditions); (ii) exclusion of extreme sleep durations (<4 h or >12 h); (iii) a finer 1-h categorical exposure (reference: 7–8 h); (iv) additional adjustment for PIR, PHQ-9, PHQ-8, and physical activity; (v) the continuous disease-count outcome modeled by weighted least squares and by survey-weighted Poisson regression; (vi) the alternative outcome definitions of Section 2.4, comprising the collapsed cardiovascular indicator, the addition of osteoporosis and of depression, the removal of arthritis, the strict non-borderline diabetes definition, and the exclusion of participants who refused or did not know any condition item; (vii) adjustment for the sleep-quality indicators, the Fried-style frailty phenotype, the FI-4 deficit index, and sedative or anxiolytic medication use, together with the analysis restricted to participants classified as non-frail on the phenotype and the deliberately over-adjusted models described in Section 2.5; (viii) reverse-causation probes that exclude participants carrying a diagnosis that plausibly causes long sleep (cancer, COPD, and heart failure, singly and jointly) and, separately, participants using sedative or anxiolytic medication, scoring PHQ-9 ≥ 10, reporting fair or poor health, hospitalized in the past year, or reporting apnea symptoms; (ix) an absolute-scale estimate, the adjusted marginal risk difference obtained by predictive margins; and (x) the E-value [31].

The E-value indexes how strongly an unmeasured confounder would need to be related to both exposure and outcome, above and beyond the measured confounders, to fully account for an observed association. Multimorbidity is common in this sample (64.0% overall and 61.0% in the 6–8 h reference stratum), and for a common outcome the odds ratio overstates the risk ratio, so an E-value computed by treating the odds ratio directly as a risk ratio is too large. We therefore converted the odds ratio to an approximate risk ratio before computing the E-value, using RR = OR/[(1 − p0) + (p0 × OR)] with p0 = 0.610, the survey-weighted outcome prevalence in the reference stratum, as recommended by VanderWeele and Ding [31]. The converted E-values are 1.51 for the point estimate and 1.20 for the lower confidence limit. For completeness we also computed the simpler square-root approximation, RR ≈ √OR, which yields 1.66 and 1.24; because that approximation gives larger and therefore less conservative bounds, we report the converted values as the primary result.

Missing data were assessed rather than assumed ignorable. Of the 3422 age-eligible participants, 424 (12.4%) were excluded from the complete-case sample. We compared included and excluded participants on age, sex, PIR, sleep duration, condition count, multimorbidity status, PHQ-9, and BMI; fitted a survey-weighted logistic model for the probability of exclusion; and applied Little’s test [32] for data missing completely at random across sleep duration, BMI, PIR, PHQ-9, age, and condition count. Excluded participants were older, slept somewhat longer, and carried more chronic conditions, and the MCAR null was rejected (χ^2^ = 267.6, df = 58, *p* < 0.001, across 16 missingness patterns), so complete-case analysis alone is not adequate justification. We therefore refitted the primary model after multiple imputation by chained equations with m = 20 imputations and Rubin’s rules for pooling [33], which returns all 3422 age-eligible participants to the analysis; the imputation was carried out independently in Python and in R with the mice package [34].

Two-sided *p*-values < 0.05 were considered statistically significant. All primary analyses were conducted in Python 3.13.6 using pandas 2.3.3, statsmodels 0.14.6, NumPy 2.2.0, SciPy 1.17.1, scikit-learn 1.6.1, and matplotlib. Because the complex-survey machinery used here, in particular delete-one-PSU jackknife variance for weighted logistic regression, jackknife confidence bands for the splines, and design-based interaction tests, was implemented directly rather than taken from an established survey package, every estimate in Table 2 was independently refitted in R 4.5.3 using the survey package [35] under both a Taylor-linearized design and a JKn jackknife replicate-weight design. Point estimates agreed to three decimal places and confidence limits to within 0.006 across the implementations, and the spline non-linearity test and the multiple imputation were reproduced in R as well. The complete Python analysis script, the R cross-validation script, a supplementary table of all fitted model coefficients, and the directed acyclic graph underlying the covariate selection are deposited as Supplementary Material, so that the analysis can be re-run rather than requested.

## 3. Results

### 3.1. Characteristics of the Study Population

Among the 2998 community-dwelling adults aged ≥60 years included in the analytic sample, the survey-weighted mean age was 69.6 years (SD = 6.7), and 54.5% were female (Table 1). Race and ethnicity were classified, and modeled, in the same five categories throughout: non-Hispanic White (74.1% of the weighted sample), non-Hispanic Black (9.2%), Hispanic (8.9%), non-Hispanic Asian (4.7%), and other (3.1%). The weighted prevalence of overweight (BMI 25–29.9) and obesity (BMI ≥ 30) was 35.6% and 41.6%, respectively; only 24 participants (weighted 0.42%) were underweight (BMI < 18.5). The weighted mean sleep duration was 7.78 h (SD = 1.58), with 8.0% reporting short sleep (<6 h), 56.8% normal sleep (6–8 h), and 35.2% long sleep (>8 h). The weighted prevalence of multimorbidity (≥2 chronic conditions) was 64.0% overall, and differed across sleep categories: 62.1% among short sleepers, 61.0% in the 6–8 h reference stratum, and 69.4% among long sleepers. All percentages reported in Table 1, including the multimorbidity row, are survey-weighted within each column.

### 3.2. Sleep Duration and Multimorbidity: Main Association

In the survey-weighted logistic regression models, long sleep duration (>8 h) was associated with higher odds of multimorbidity in both the crude model (OR = 1.45, 95% CI: 1.09–1.91, *p* = 0.012) and the primary adjusted model controlling for age, sex, race/ethnicity, education, BMI, and smoking (aOR = 1.41, 95% CI: 1.08–1.85, *p* = 0.014; Table 2). Short sleep duration was not associated with multimorbidity in either model (aOR = 0.92, 95% CI: 0.58–1.47, *p* = 0.726). Expressed on the absolute scale by survey-weighted g-computation from the primary model, the adjusted prevalence of multimorbidity was 68.7% among long sleepers and 61.7% in the 6–8 h reference stratum, a difference of 7.0 percentage points (95% CI 1.7 to 12.2). This is a moderate difference on a background prevalence above 60%, and we interpret it as such throughout.

Model 3, which adds the PHQ-9 depressive symptom score, attenuated the long-sleep association (aOR = 1.32, 95% CI: 0.95–1.82, *p* = 0.095). It is treated as a sensitivity analysis rather than as a main result, because sleep duration, depressive symptoms, and chronic conditions were all measured at a single timepoint and we therefore cannot distinguish whether depression confounds, mediates, or shares common causes with the outcome; conditioning on a mediator would bias an estimate of the total effect.

**Figure 2:**
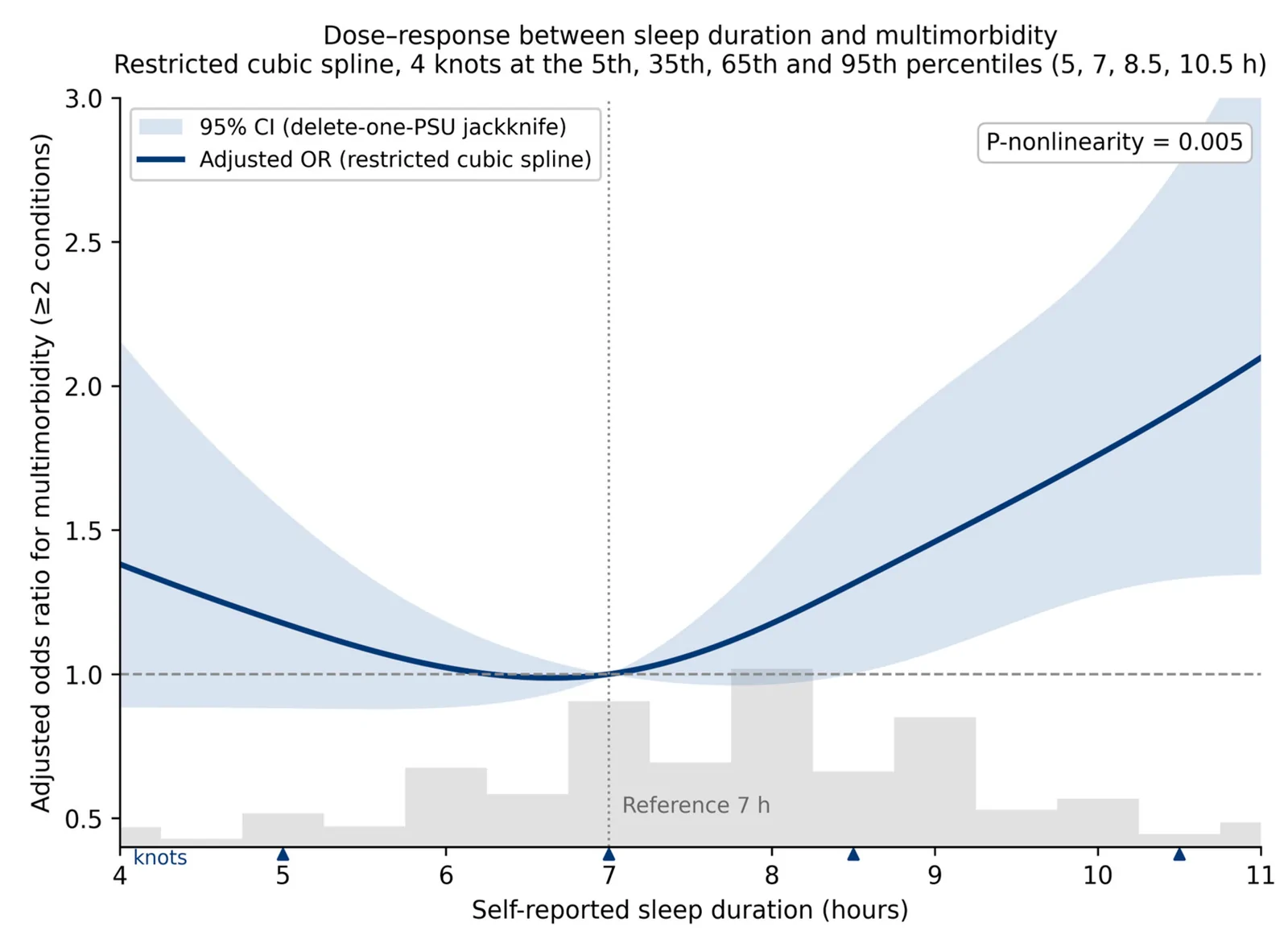
Restricted cubic spline modeling of the dose–response relationship between sleep duration and multimorbidity (≥2 chronic conditions). Four knots placed at the canonical Harrell percentiles (5th, 35th, 65th, 95th; 5.0, 7.0, 8.5, 10.5 h). Reference: 7 h. Adjusted for age, sex, race/ethnicity, education, BMI, and smoking. Shaded area = 95% CI (delete-one-PSU jackknife). P-nonlinearity = 0.005 by jackknife and 0.020 by Taylor linearization.

The dose–response analysis using restricted cubic splines confirmed a non-linear, J-shaped relationship between sleep duration and multimorbidity (Figure 2). Knots were placed at the canonical Harrell percentiles (5th, 35th, 65th, and 95th, corresponding to 5.0, 7.0, 8.5, and 10.5 h). With 7 h as the reference, the adjusted odds of multimorbidity were essentially flat between 6 and 8 h (aOR at 8 h = 1.18, 95% CI 0.97–1.43) and then rose progressively: aOR = 1.46 (1.08–1.97) at 9 h and aOR = 1.76 (1.28–2.43) at 10 h. The apparent elevation at short sleep durations did not reach statistical significance at any point. Non-linearity was significant under both variance estimators, although the two do not agree closely: P-nonlinearity = 0.005 under the delete-one-PSU jackknife used for the primary analysis and *p* = 0.020 under Taylor-series linearization in R (Table S14). The J-shape itself was robust to knot specification, with P-nonlinearity of 0.038, 0.005, and 0.012 across canonical 3-, 4-, and 5-knot models and 0.005 under an alternative placement at the 5th, 27.5th, 72.5th, and 95th percentiles (Table S13).

A more granular 1-h categorical analysis (reference: 7–8 h) located the excess risk precisely. Odds of multimorbidity were not elevated below the reference band (<6 h: aOR = 0.96, 95% CI 0.58–1.58; 6–7 h: aOR = 1.16, 0.68–1.97), and they were not elevated in the hour immediately above it (>8 to <9 h: aOR = 1.08, 95% CI 0.67–1.73, *n* = 229). The elevation was confined to participants reporting ≥9 h (aOR = 1.65, 95% CI 1.22–2.25, *p* = 0.002, *n* = 833). Because responses were recorded to the nearest half-hour and 8.0 h falls within the reference band, the band immediately above the reference is >8 to <9 h rather than 8–9 h. The excess risk is therefore specific to the ≥9 h range and not to the >8 h category as a whole, which combines the 833 participants reporting ≥9 h with 229 whose odds are close to the referent and so dilutes the signal.

Modeling the disease count as a continuous outcome corroborated the categorical finding. Long sleepers had on average 0.19 more chronic conditions than normal sleepers by weighted least squares (β = 0.185, 95% CI 0.020–0.350, *p* = 0.030), and a survey-weighted Poisson model gave an incidence rate ratio of 1.09 (95% CI 1.01–1.17, *p* = 0.029).

### 3.3. Subgroup Analyses with Tests of Interaction

**Figure 3:**
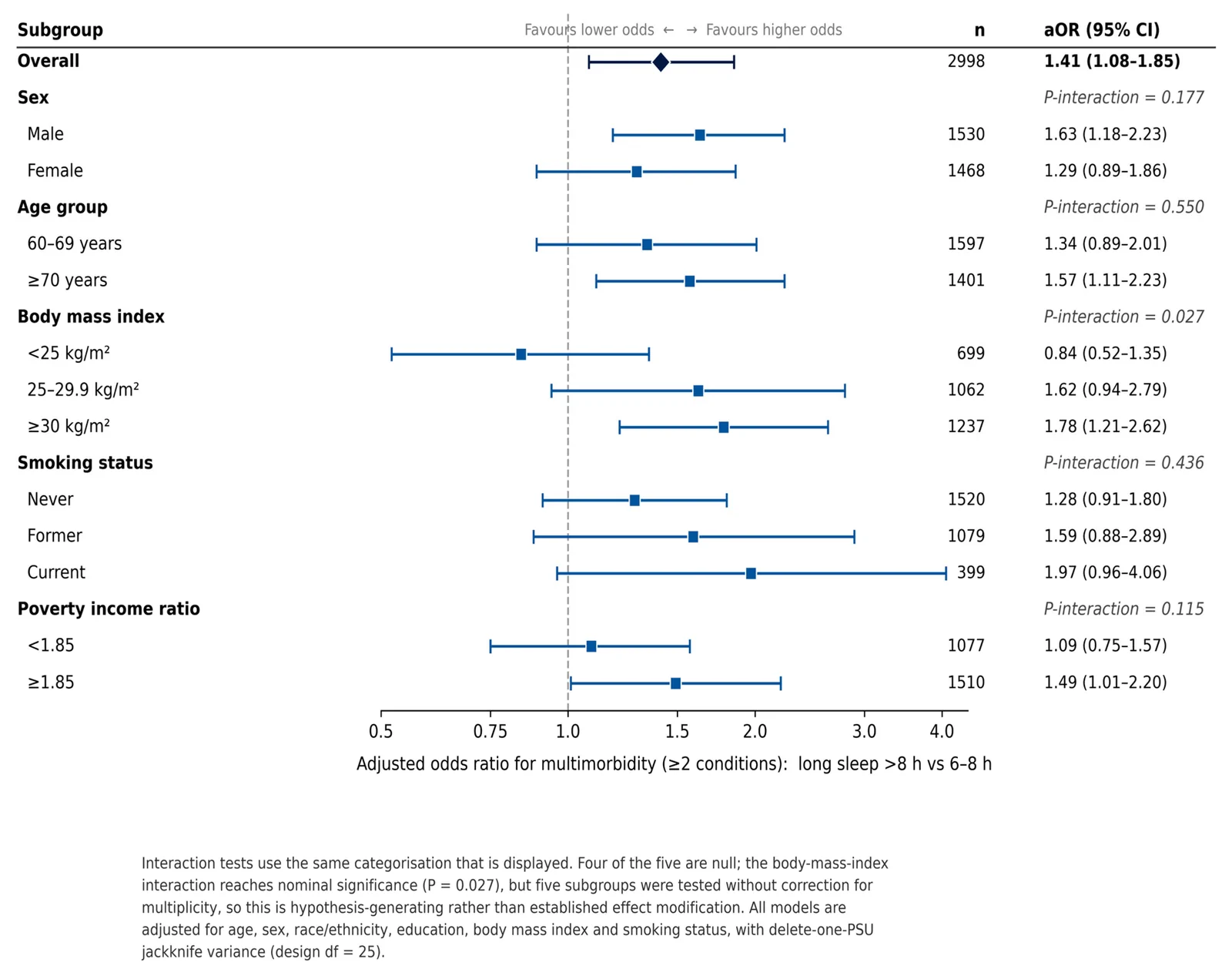
Forest plot of the adjusted association between long sleep duration (>8 h vs. 6–8 h) and multimorbidity (≥2 chronic conditions), stratified by subgroup, with formal tests of interaction. P-interaction was computed by Wald test on the multiplicative interaction terms using the same categorization that is displayed. Four of the five interaction tests were non-significant; the test across BMI categories reached *p* = 0.027, but it is not robust to entering BMI continuously (*p* = 0.450) and was one of five uncorrected tests, so it should be read as hypothesis-generating. All models adjusted for age, sex, race/ethnicity, education, BMI, and smoking.

Stratum-specific estimates are shown in Figure 3. The point estimate exceeded 1 in eleven of the twelve strata examined. The association was larger and reached significance among males (aOR = 1.63, 95% CI 1.18–2.23, *p* = 0.004), adults aged ≥70 years (aOR = 1.57, 1.11–2.23, *p* = 0.013), participants with obesity (aOR = 1.78, 1.21–2.62, *p* = 0.005), and those in the higher-income stratum (PIR ≥1.85; aOR = 1.49, 1.01–2.20, *p* = 0.043). Estimates were above the null but not statistically significant among females (aOR = 1.29, 0.89–1.86), adults aged 60–69 (1.34, 0.89–2.01), participants who were overweight (1.62, 0.94–2.79), never (1.28, 0.91–1.80), former (1.59, 0.88–2.89) and current (1.97, 0.96–4.06) smokers, and the lower-income stratum (1.09, 0.75–1.57). The one exception was the normal-range BMI stratum (BMI < 25, *n* = 699), in which the point estimate fell below the null (aOR = 0.84, 95% CI 0.52–1.35, *p* = 0.459).

Formal tests of effect modification were computed on the same categorizations that are displayed in Figure 3, so that the test and the figure refer to the same quantity. Four of the five tests did not approach significance (P-interaction: sex = 0.177, age = 0.550, smoking = 0.436, PIR = 0.115), but the test across the three BMI categories did (P-interaction = 0.027), driven by the contrast between the BMI < 25 stratum (aOR = 0.84) and the overweight and obese strata (1.62 and 1.78). Three considerations argue against reading this as established effect modification. First, it is not robust to how adiposity is entered: tested with BMI as a continuous moderator the same interaction gives *p* = 0.450. Second, five subgroups were tested and the BMI result does not survive correction for multiplicity (Bonferroni-adjusted *p* = 0.137; Benjamini–Hochberg q = 0.137), so one *p*-value near 0.03 is close to what would be expected under a global null. Third, the BMI < 25 stratum is the smallest of the three and its confidence interval (0.52–1.35) is compatible with estimates on either side of the null. We therefore report the BMI interaction as hypothesis-generating and do not propose any BMI-specific interpretation of the sleep–multimorbidity association on this basis. The remaining stratum-level differences are best read as descriptive heterogeneity in point estimates rather than as statistically distinguishable effect modification.

### 3.4. Sensitivity Analyses

The direction of the primary association was consistent across sensitivity analyses, although its magnitude and statistical significance were sensitive to the outcome definition and to the extent of covariate adjustment (Table 3). Under more stringent multimorbidity definitions the association attenuated to borderline non-significance at the ≥3 threshold (aOR = 1.30, 95% CI 0.99–1.72, *p* = 0.061) and to non-significance at the ≥4 threshold (aOR = 1.12, 0.80–1.57, *p* = 0.488), consistent with the J-shaped pattern observed in the spline model. Exclusion of extreme sleep durations (<4 h or >12 h; *n* = 2917) produced essentially identical estimates (aOR = 1.40, 1.07–1.84, *p* = 0.018). Additional adjustment for PIR, PHQ-9, and vigorous physical activity (*n* = 2415) attenuated the association to non-significance (aOR = 1.34, 0.91–1.96, *p* = 0.129); as noted above, with cross-sectional data this attenuation could reflect confounding, mediation, or shared upstream causes, and we draw no mechanistic conclusion from it.

Varying the condition list left the estimate broadly stable (Table S9). Collapsing congestive heart failure, coronary heart disease, and myocardial infarction into a single cardiovascular indicator, which addresses the possibility that one cardiovascular trajectory is counted up to three times in the same participant, gave aOR = 1.42 (1.09–1.86, *p* = 0.012); 521 participants reported at least one of the three diagnoses, of whom 302 reported one, 156 reported two, and 63 reported all three. Adding osteoporosis and PHQ-9-defined depression, giving a 13-condition definition at a weighted prevalence of 67.6%, gave aOR = 1.36 (1.08–1.70, *p* = 0.010). Removing arthritis, the most prevalent single condition (weighted 52.6%; Table S1), attenuated the estimate but left it significant (aOR = 1.31, 1.02–1.68, *p* = 0.034), and restricting diabetes to diagnosed non-borderline disease left it essentially unchanged (aOR = 1.39, 1.05–1.84, *p* = 0.023). Excluding the 77 participants with a refused or don’t-know response on any condition item also left it unchanged (aOR = 1.40, 1.06–1.85, *p* = 0.021, *n* = 2921), as did separating underweight (BMI < 18.5) into its own BMI category (aOR = 1.42, 1.08–1.85, *p* = 0.014).

Analyses directed at reverse causation are summarized in Table S2. Excluding participants reporting each of the three conditions most likely to lengthen sleep in their own right left the estimate intact when done singly: cancer (aOR = 1.38, 1.06–1.80, *n* = 2357), chronic obstructive pulmonary disease (1.44, 1.04–1.98, *n* = 2547), and congestive heart failure (1.40, 1.06–1.84, *n* = 2770). Excluding all three jointly reduced the estimate to non-significance (aOR = 1.31, 0.96–1.78, *p* = 0.083, *n* = 1914), but this exclusion also removes 1084 participants, roughly a third of the sample, and the weighted prevalence of the outcome falls from 64.0% to 48.4%, so the loss of significance cannot be separated from the loss of outcome events and precision. Excluding participants taking a sedative or anxiolytic medication (aOR = 1.30, 0.97–1.74), those with PHQ-9 ≥ 10 (1.33, 0.96–1.85), those in fair or poor self-rated health (1.29, 0.94–1.77), and those hospitalized in the past year (1.31, 1.00–1.73) each moved the estimate downward by a similar amount without reversing it, whereas excluding participants reporting witnessed snorting or breathing pauses did not (1.40, 1.07–1.83). These analyses do not eliminate reverse causation. What they show is that no single condition or marker of illness severity accounts for the association, and that the association is somewhat weaker in progressively healthier subsamples.

Two further checks bear on the credibility of the primary estimate. First, the 424 age-eligible participants excluded for missing covariate data (12.4%) were on average older and had more chronic conditions than those retained, and Little’s test [32] rejected a missing-completely-at-random mechanism (chi-square = 267.6, df = 58, *p* < 0.001; Table S10). Repeating the primary model under multiple imputation by chained equations (m = 20) across all 3422 age-eligible participants returned essentially the complete-case estimate (aOR = 1.39, 95% CI 1.08–1.79, *p* = 0.012), as did an independent implementation in R (1.39, 1.03–1.89, *p* = 0.038), whose wider interval reflects a different treatment of the survey design across imputed datasets (Table S11). Second, every estimate in Table 2 was refitted in R using the survey package [35] under both Taylor-series linearization and a JKn jackknife replicate-weight design; point estimates agreed to three decimal places, confidence limits agreed to within 0.006, and the R design returned the same 25 design degrees of freedom (49 pseudo-PSUs minus 24 pseudo-strata). Agreement was not universal, and we report the exceptions rather than summarizing the comparison as uniform: the test for spline non-linearity differs materially between variance estimators (*p* = 0.005 by jackknife, *p* = 0.020 by Taylor linearization), and the two multiple-imputation implementations differ in interval width as described above. The complete comparison, including the rows that disagree, is given in Table S14.

The fully adjusted model (Table 3, row A6) deserves a separate note, because its attenuation to non-significance could be read as evidence that the association is fragile. Adding the poverty income ratio, PHQ-9 score, and physical activity reduces the analytic sample from 2998 to 2415, so the attenuation confounds adjustment with loss of information. Refitting the primary model on that same complete-case subsample, without the three additional covariates, gives aOR = 1.42 (95% CI 1.00–2.02, *p* = 0.049): the point estimate is unchanged and the interval widens through the 19% loss of sample. Adding the three covariates then moves the estimate only from 1.42 to 1.34 (0.91–1.96). The three covariates therefore account for the movement in the point estimate, while the loss of sample accounts for the loss of significance: the interval widens enough that a similar estimate no longer excludes the null (Table S18).

Applying the E-value formula directly to the odds ratio gives 2.18 for the point estimate and 1.37 for the lower confidence limit. Because multimorbidity is common in this sample (weighted prevalence 64.0% overall and 61.0% in the 6–8 h reference stratum), the odds ratio overstates the corresponding risk ratio, and an E-value computed on the odds-ratio scale therefore overstates the strength of confounding required to explain the association away. Converting the odds ratio to an approximate risk ratio using the baseline risk in the reference stratum (p0 = 0.610) gives RR = 1.129 and a corrected E-value of 1.51 for the point estimate and 1.20 for the lower confidence limit. The simpler square-root-of-odds-ratio approximation gives 1.66 and 1.24, which are larger than the values obtained by the exact conversion and therefore less conservative, so we report the converted values as primary and the approximation alongside them (Table S12).

The corrected bound is modest, and we state its implication plainly. An unmeasured confounder associated with both long sleep and multimorbidity by a risk ratio of about 1.5, above and beyond the measured covariates, would suffice to account for the point estimate, and one of about 1.2 would suffice to move the lower confidence limit to the null. Undiagnosed obstructive sleep apnea, sedative or anxiolytic medication use, and frailty are each plausible at that magnitude in an older population. We therefore interpret the E-value as a transparency bound that the observed association does not clear by a wide margin, rather than as evidence that the association is unconfounded; and it does not address reverse causation or temporal ambiguity, which remain separate threats in this cross-sectional design.

### 3.5. Sleep Quality and Frailty

Nine hours of consolidated sleep is not the same exposure as nine hours of fragmented sleep, so we examined four sleep-quality indicators from the same questionnaire (Tables S3–S5). Poor sleep quality was common and only modestly patterned by duration: the composite indicator (a complaint of trouble sleeping, frequent daytime sleepiness, or witnessed snorting or breathing pauses) was present in 49.2% of the weighted sample, in 45.7% of those sleeping 6–8 h and in 51.3% of those sleeping >8 h. Adjusting the primary model for each indicator singly changed the long-sleep estimate little (aOR range 1.38 to 1.42), and adjusting for all four simultaneously gave aOR = 1.37 (95% CI 0.99–1.89, *p* = 0.060, *n* = 2565), an interval that includes the null largely because 433 participants are lost to item non-response.

Entering duration and quality jointly, with normal 6–8 h sleep of good quality as the reference (*n* = 914), showed that both carry information. Normal-duration sleep of poor quality was associated with multimorbidity (aOR = 2.44, 95% CI 1.70–3.49, *n* = 722), as was long sleep of good quality (aOR = 1.47, 1.04–2.09, *n* = 551), and the two together carried the highest odds (aOR = 3.00, 2.23–4.05, *n* = 511). We report this pattern descriptively and draw no mediation inference from it: exposure, quality, and outcome were all recorded at a single visit, and this design cannot establish that quality lies on, or off, a pathway from duration to disease.

Frailty was examined with two measures of deliberately different construction (Tables S6 and S7). The primary measure is a partial Fried phenotype comprising unintentional weight loss of ≥5% in the past year, absence of moderate or vigorous recreational activity, and exhaustion; grip strength and gait speed were not collected in this cycle, so three of Fried’s five criteria are available. None of the three is a measure of health-care utilization or of medication. The exhaustion criterion is nevertheless measured by a PHQ-9 item asking about tiredness and low energy, which is plausibly a consequence of the exposure itself, so we also report a two-component version restricted to weight loss and inactivity, neither of which is sleep-derived. In the weighted sample, 39.4% had none of the three components, 43.7% had one, 14.2% had two, and 2.6% had all three, and the mean component score was higher among long sleepers than among normal sleepers (0.85 vs. 0.74). The phenotype correlates 0.22 with the chronic-condition count, less than half the correlation of 0.47 observed for an 11-item deficit-accumulation index built from the same data.

The association was not explained by frailty as measured this way. Adding the phenotype to the primary model gave aOR = 1.38 (95% CI 1.00–1.90, *p* = 0.049, *n* = 2732), and adding a deficit index restricted to the four items that are not consequences of accumulated diagnoses gave aOR = 1.38 (1.03–1.85, *p* = 0.031, *n* = 2986). The most informative analysis is a restriction rather than an adjustment: among the 2205 participants who were non-frail on the phenotype, that is with fewer than two of the three components, the association was 1.42 (1.03–1.97, *p* = 0.034). If frailty were generating the association, restricting the sample to non-frail participants should have weakened it; instead it was unchanged. Adjustment for a sedative or anxiolytic medication, which was reported by 15.2% of long sleepers compared with 9.3% of normal sleepers, likewise left the estimate essentially intact (aOR = 1.37, 1.05–1.79, *p* = 0.021; Table S8).

Two adjustments did remove statistical significance, and we report them while stating explicitly that they are over-adjustments. Adding the full 11-item deficit index gave aOR = 1.18 (0.84–1.65, *p* = 0.326), and adding the number of prescription medications gave aOR = 1.19 (0.84–1.68, *p* = 0.318). Five of the eleven deficits, namely polypharmacy, four or more health-care visits, hospitalization in the past year, fair or poor self-rated health, and osteoporosis, are consequences of having accumulated chronic diagnoses rather than causes of accumulating them, and each is strongly associated with the outcome in this sample (odds ratios of 7.06, 3.62, 4.34, 3.76, and 1.70 respectively; Table S6). The index as a whole correlates 0.47 with the condition count, and adjusting for those five items alone reproduces almost the entire attenuation (aOR = 1.22, 0.86–1.73). Conditioning on them removes part of the outcome itself, so the attenuated estimates quantify how much of multimorbidity is captured by its own downstream markers and cannot be read as evidence that the sleep association is confounded by frailty. No conclusion in this paper rests on them.

Adjustment for the two-component phenotype left the association unchanged (aOR = 1.43, 95% CI 1.08–1.89, *p* = 0.013, *n* = 2937); adding the exhaustion criterion, which is partly a consequence of sleep itself, gave the more conservative aOR = 1.38 (1.00–1.90, *n* = 2732). Both are compatible with the unadjusted estimate of 1.41.

## 4. Discussion

### 4.1. Principal Findings

In this nationally representative analysis of U.S. older adults from the NHANES 2017–March 2020 pre-pandemic cycle, long sleep duration (>8 h), but not short sleep duration, was associated with higher odds of multimorbidity (aOR = 1.41, 95% CI: 1.08–1.85), a difference of 7.0 percentage points (1.7 to 12.2) on the absolute scale. Restricted cubic spline modeling described a non-linear, J-shaped dose–response, and the 1-h analysis placed the excess risk entirely at ≥9 h (aOR = 1.65, 1.22–2.25); the hour immediately above the reference band, >8 to <9 h, showed no elevation at all (aOR = 1.08, 0.67–1.73). Point estimates exceeded 1 in eleven of the twelve subgroup strata, the exception being participants with BMI < 25, and the interaction across BMI categories was the only one of five tests to reach conventional significance (*p* = 0.027), which we treat as hypothesis-generating rather than as established effect modification.

The association was not explained by frailty as we were able to measure it. It was not materially attenuated by adjustment for a partial Fried phenotype (aOR = 1.38, 1.00–1.90) and after adjustment for a deficit index restricted to items that are not consequences of accumulated diagnoses (1.38, 1.03–1.85), and, most informatively, it was preserved among participants who were non-frail on that phenotype (1.42, 1.03–1.97). Had frailty been generating the association, restricting the analysis to non-frail participants should have weakened it; it did not. Adjustment for the full 11-item deficit index (1.18) or for the number of prescription medications (1.19) did remove significance, but both condition on consequences of having accumulated chronic diagnoses and are therefore over-adjustments rather than informative controls, and we build nothing on them.

What does move the estimate is the definition of the outcome and the breadth of covariate adjustment: the association attenuates at the ≥3 and ≥4 condition thresholds, and after adding depressive symptoms, income, and physical activity. Because all measurements come from a single visit, we cannot determine whether those covariates confound, mediate, or share common causes with the outcome. Taken with a corrected E-value of 1.51 and a design that cannot order sleep and disease in time, the honest summary is a moderate, specification-sensitive association that is real in these data but not securely causal.

### 4.2. Comparison with Prior Literature

Long sleep duration has been associated with a range of single conditions in older adults, including type 2 diabetes [16], cardiovascular disease [6,36], cognitive decline [37], and mortality [38,39]. By taking multimorbidity as the outcome, we extend this work to the cumulative count of chronic conditions, which is more clinically relevant for older patients who rarely present with a single disease [40].

The J-shaped pattern we observed is consistent with prior meta-analyses [41,42], and the concentration of excess risk at ≥9 h aligns with the National Sleep Foundation’s 7–8 h recommendation for older adults [22]. Our ≥9 h signal is also concordant with prospective cohort evidence: in the Whitehall II study, long sleep (≥9 h) measured at age 60 (HR = 1.54, 95% CI 1.15–2.06) and at age 70 (HR ≈ 1.51) predicted higher risk of incident multimorbidity, whereas at age 50, when long sleepers were few (*n* = 77), the association was not robust [14]. That long-sleep risk emerged prospectively only at older ages is consistent with our focus on adults aged ≥60 and lends temporal support to the ≥9 h threshold.

Three qualifications keep that comparison in proportion. First, the Whitehall II investigators themselves characterized their long-sleep findings as less robust than their short-sleep findings, so the prospective evidence for the exposure we study is the weaker half of that study’s result. Second, their multimorbidity outcome comprised 13 conditions to our 11, and a different condition list changes both the prevalence of the outcome and which participants cross the threshold; our own outcome-definition analyses show that this alone can move the estimate by an appreciable amount. Third, Whitehall II sampled British civil servants, an occupationally and socioeconomically healthier cohort than a general older U.S. population, and healthier cohorts are exactly where long sleep is least likely to be acting as a marker of established disease. The concordance is therefore partial rather than confirmatory, and it does not by itself establish causality.

Our null result for short sleep contrasts with the robust short-sleep associations reported in Whitehall II (≤5 h at ages 50, 60, and 70) [14] and with U-shaped patterns in some general-population studies [6,38,43,44]. The most likely explanations are the low prevalence of short sleep in our older sample (weighted 8.0%, unweighted *n* = 299) and the limited power in that stratum once the complex design is accounted for (overall design effect ≈ 2.5, effective *n* ≈ 1200), together with reliance on self-report; the short-sleep null is therefore better read as absence of evidence than as evidence of absence. The attenuation we observed after adjusting for depressive symptoms fits with prior work linking long sleep and depression in older adults [45,46]. Because the PHQ-9 itself contains a sleep item, we repeated this adjustment using an 8-item score that excluded that item and obtained a near-identical estimate (aOR = 1.31, 95% CI 0.94–1.82), indicating that the attenuation reflects genuine shared variance with depression rather than mechanical over-adjustment. The temporal direction still cannot be determined with cross-sectional data.

### 4.3. Plausible Biological and Behavioral Mechanisms

Multiple biological and behavioral pathways could link long sleep to multimorbidity, although our data cannot adjudicate among them. Long sleep duration has been associated with elevated CRP and IL-6 in older adults [47,48]; both markers independently predict cardiometabolic and musculoskeletal disease. Aberrant sleep duration has also been linked to metabolic syndrome in NHANES adults [49], providing biological plausibility for its association with cumulative chronic disease burden. Long sleep is also frequently a marker of fragmented or low-quality sleep [50], or of unrecognized conditions such as obstructive sleep apnea, anemia, or early cognitive decline [11,51]. Reduced daytime physical activity among long sleepers is another plausible route, and is consistent with the partial attenuation we observed after adjusting for vigorous activity [52]. Depressive symptoms are similarly tied to both sleep and chronic disease, and could plausibly act as confounder, mediator, or downstream consequence [53].

We emphasize that cross-sectional data cannot distinguish among these possibilities. The most defensible reading is clinical rather than mechanistic. In older adults, prolonged time in bed, physical inactivity, and depressive symptoms rarely occur in isolation; they co-occur as a recognizable clinical picture, described variously as apathy, motivational withdrawal, or the behavioral expression of early frailty or of subclinical neurodegenerative change. Our own data show the same clustering: participants sleeping >8 h were more likely than those sleeping 6–8 h to report no moderate or vigorous recreational activity, higher depressive symptom scores, more prescription medications, and at least one indicator of poor sleep quality, and their mean Fried-phenotype component score was correspondingly higher.

Read this way, depressive symptoms are better understood as a shared manifestation of that picture, or as an upstream driver of it, than as a nuisance variable whose removal purifies the sleep estimate. The attenuation we observed when PHQ-9, income, and physical activity were entered together is what one should expect when correlated features of a single syndrome are adjusted for simultaneously, and it identifies none of them as causal. The same caution applies in the opposite direction, and our frailty analyses make the point concretely: adjusting away every correlate of accumulated disease, including its consequences, drives any such estimate toward the null without establishing anything about mechanism. Distinguishing an upstream driver from a parallel manifestation will require longitudinal designs with repeated measurement of sleep, mood, activity, and physical function, and ultimately intervention studies; a single visit cannot do it.

### 4.4. Strengths and Limitations

This study has several important strengths. First, the use of NHANES provides a large, nationally representative sample of community-dwelling U.S. older adults, with rigorous quality control and standardized assessment of chronic conditions. Second, our analytical approach incorporated the full complex survey design, including stratification, primary sampling units, and weights, ensuring valid inference for the U.S. population, and the design degrees of freedom are traced explicitly to the released variance structure for this cycle. Third, we employed multiple complementary analytical strategies (categorical, 1-h categorical, continuous, and spline-based) to characterize the dose–response relationship, and we report the primary contrast on the absolute as well as the relative scale. Fourth, we adopted methodologically rigorous approaches to subgroup analysis by reporting formal interaction tests, computed on the categorizations actually displayed, alongside stratum-specific estimates, addressing a well-recognized but frequently overlooked source of overinterpretation in observational research [18]. Fifth, the frailty analyses were designed to avoid the over-adjustment that a deficit-accumulation index invites when its items are themselves consequences of the outcome, and the key frailty result is a restriction to non-frail participants rather than an adjustment, which conditions on the phenotype in a different functional form rather than escaping conditioning altogether. Sixth, the E-value was computed on the risk-ratio scale appropriate to a common outcome, and the value obtained on the odds-ratio scale is shown alongside for comparison. Finally, the complete Python and R analytic code is deposited rather than available on request, the headline estimates were reproduced under an independent implementation in R, and the points at which the two implementations disagree are reported rather than smoothed over.

This study also has limitations. First, the cross-sectional design precludes causal inference. Reverse causation, whereby multimorbidity promotes longer time in bed through pain, deconditioning, or medication, or promotes the perception of long sleep, is the primary alternative explanation and cannot be excluded. Our reverse-causation analyses narrow it without closing it: excluding cancer, chronic obstructive pulmonary disease, or heart failure singly left the estimate essentially unchanged, but excluding all three jointly reduced it to 1.31 (0.96–1.78), and exclusions based on sedative or anxiolytic use, clinically significant depressive symptoms, fair or poor self-rated health, and recent hospitalization each pulled the estimate toward the null. The E-value bounds unmeasured confounding but not this temporal ambiguity, and the concordant prospective ≥9 h findings in Whitehall II [14] offer partial rather than definitive reassurance.

Second, sleep duration was self-reported using a single-item questionnaire, which may be subject to recall bias and correlates only modestly with objective polysomnographic or actigraphic measures [54]. A self-reported duration conflates time in bed with sleep obtained, a distinction that matters most in exactly the group that drives our result. We were able to add four sleep-quality indicators, but they are also self-reported and were missing for a non-trivial minority.

Third, the outcome was a count of eleven heterogeneous, self-reported physician-diagnosed conditions given equal weight. It may underestimate undiagnosed disease and is open to surveillance bias, whereby greater health-care contact yields more recorded diagnoses. Two specific features of the count deserve mention. Arthritis was highly prevalent, and excluding it attenuated but did not abolish the association (aOR = 1.31). Congestive heart failure, coronary heart disease, and myocardial infarction were counted separately although they frequently reflect a single underlying cardiovascular trajectory, so the count can be inflated for cardiovascular patients relative to participants with an equally severe but singly coded condition: 219 of the 521 participants with any of these diagnoses reported two or three of them. Collapsing the three into one indicator left the estimate unchanged (aOR = 1.42, 1.09–1.86), which bounds the impact of this inflation but does not remove the general concern about equal weighting.

Fourth, residual confounding cannot be excluded, and the corrected E-value shows that it need not be large. An unmeasured confounder related to both long sleep and multimorbidity by a risk ratio of about 1.5 would suffice to explain the point estimate, and about 1.2 would suffice for the lower confidence limit. Undiagnosed obstructive sleep apnea remains the most plausible candidate, since NHANES ascertains only symptoms and not a sleep study, and sedative or anxiolytic medication use and clinical frailty are two further candidates that are tied to both increased time in bed and disease burden. We measured the latter two directly and adjustment for either left the primary estimate substantially intact, which constrains but does not eliminate their potential contribution, since both are measured with error.

Fifth, our frailty measurement is partial. The Physical Functioning questionnaire file was not carried into the 2017–March 2020 pre-pandemic combined release, so activities-of-daily-living, instrumental-activities, and mobility items are unavailable, and grip strength and gait speed were not collected; the record of this availability check is deposited with the analytic code. Our phenotype therefore captures three of Fried’s five criteria and a standard 36- to 49-item deficit index could not be constructed. A more complete frailty measure could attenuate the association further than ours did. The converse error is equally real and is worth stating explicitly, because our own first pass made it: a deficit-accumulation index that includes polypharmacy, health-care visits, hospitalization, self-rated health, and osteoporosis conditions on consequences of accumulated diagnoses, and adjusting for it removes part of the outcome rather than a confounder. The resulting attenuation to 1.18, and the parallel attenuation to 1.19 on adjustment for medication count, are reported here for transparency and should not be read as evidence against the association.

Sixth, our findings derive from U.S. data and may not generalize to populations with different demographic compositions or health-care systems; even within the U.S., recent nationally representative data show substantial sociodemographic and structural disparities in short sleep that could shape population-specific risk profiles [55]. Related to this, only 24 participants (weighted 0.42%) were underweight, too few to model as a separate stratum with any precision. The BMI < 25 category therefore combines underweight with normal weight, which is not ideal: in older adults underweight may itself mark frailty, sarcopenia, or undiagnosed disease, and could act in the opposite direction from overweight and obesity. Separating it as a fourth category left the primary estimate essentially unchanged (aOR = 1.42 versus 1.41), but the subgroup finding in the BMI < 25 stratum should be read with this heterogeneity in mind.

Seventh, the primary association attenuated to non-significance under stricter multimorbidity thresholds (≥3 and ≥4 conditions) and after additional adjustment for depressive symptoms, family income, and physical activity, although it persisted when extreme sleep values were excluded, when disease count was modeled continuously, and under alternative condition lists. The estimate is therefore moderate and specification-sensitive rather than definitive, and part of the attenuation in the fully adjusted model (*n* = 2415) reflects reduced sample size as well as additional adjustment.

Finally, the primary analysis is a complete-case analysis excluding 12.4% of age-eligible participants, and Little’s test [32] rejected a missing-completely-at-random mechanism. Multiple imputation across all 3422 age-eligible participants returned essentially the same estimate in both a Python and an R implementation, which is reassuring, but imputation corrects only for data missing at random given the imputation model, and the excluded participants were older and sicker than those retained, so a residual selection effect in the direction of the null cannot be ruled out.

### 4.5. Interpretation and Directions for Future Research

We make no clinical recommendation, and these data support none. A cross-sectional survey can show that habitual long sleep and accumulated chronic disease occur together in older U.S. adults; it cannot show that either produces the other, and nothing here indicates that changing how long an older adult sleeps would change how much disease that person accumulates. The most that can be said is that long sleep, and specifically ≥9 h, travels with a heavier burden of diagnosed disease, which is consistent with long sleep being a marker of that burden rather than a cause of it. We are not proposing that sleep duration be used as a screening trigger, and we would caution against reading a 7 percentage point difference in a common condition as a basis for changing practice.

Habitual long sleep might also serve as a prompt to initiate a Comprehensive Geriatric Assessment, focusing on polypharmacy, mood, mobility, and sleep apnea screening, rather than as a reason to advise patients to spend less time in bed. We think that is a reasonable hypothesis and we set it out as one. Our data are consistent with it only descriptively: among participants reporting >8 h, 51.3% had at least one indicator of poor sleep quality, 15.2% were taking a sedative or anxiolytic medication, and 46.0% reported five or more prescription medications, compared with 45.7%, 9.3%, and 32.7% among those sleeping 6–8 h. Those domains are precisely the ones a Comprehensive Geriatric Assessment covers. But our data cannot show that an assessment triggered by sleep duration would identify problems earlier than usual care, would change management, or would benefit patients, and the group reporting ≥9 h is not a small minority of this population (*n* = 833 of 2998), so the resource implications of treating it as a trigger are not trivial. Whether long sleep carries incremental case-finding value beyond what a clinician already knows about a patient is an empirical question that this study raises and does not answer, and it would be answered by a prospective study of assessment yield, not by a cross-sectional association.

We likewise propose no subgroup-specific interpretation. The interaction across BMI categories was the only one of five uncorrected tests to reach significance and was not robust to entering BMI continuously, so it is a hypothesis about adiposity and sleep worth testing in an independent sample, not a basis for differentiating patients now. The priorities for future work follow from what this design cannot do. Prospective cohorts with repeated sleep measurement are needed to order sleep and disease in time. Actigraphy or polysomnography would separate time in bed from sleep obtained and would identify sleep-disordered breathing. The frailty phenotype needs to be measured in full, including the grip strength and gait speed that were unavailable in this cycle. Ultimately the question calls for intervention studies that establish whether sleep in older adults is a modifiable target at all, or only a window onto health that is already declining.

## 5. Conclusions

In this nationally representative sample of U.S. adults aged 60 and over, long sleep duration, and specifically nine hours or more, was associated with higher odds of multimorbidity along a non-linear, J-shaped dose–response; the hour immediately above the conventional >8 h cut-point showed no elevation. On the absolute scale the association was moderate, at 7.0 percentage points (95% CI 1.7 to 12.2), and its direction was consistent in eleven of the twelve subgroup strata. It was not explained by frailty as we were able to measure it: it was not materially attenuated by adjustment for the frailty phenotype and for a deficit index confined to items that are not consequences of accumulated diagnoses, and it was preserved among participants who were non-frail on that phenotype. It nonetheless attenuated under stricter multimorbidity definitions and under fuller covariate adjustment, a corrected E-value of 1.51 indicates that moderate unmeasured confounding would suffice to account for it, and the cross-sectional design cannot exclude reverse causation. We therefore cannot say whether long sleep contributes to multimorbidity, reflects it, or both, and we do not treat it as an actionable risk factor. What these data do support is a more modest reading: habitual long sleep in older adults marks an accumulated burden of chronic disease, and is worth noting in the clinical assessment of this group.

## Figures and Tables

**Table 1:** Characteristics of the study population (*n* = 2998), overall and stratified by sleep duration category. NHANES 2017–March 2020.

Characteristic	Overall (*n* = 2998)	Short < 6 h (*n* = 299)	Normal 6–8 h (*n* = 1637)	Long >8 h (*n* = 1062)
Age, years (mean ± SD)	69.6 ± 6.7	68.4 ± 6.7	68.9 ± 6.5	70.9 ± 6.7
Female, *n* (%)	1468 (54.5)	124 (47.4)	771 (50.4)	573 (62.7)
Race/Ethnicity, *n* (%)				
Non-Hispanic White	1281 (74.1)	95 (64.3)	691 (74.8)	495 (75.2)
Non-Hispanic Black	802 (9.2)	119 (16.3)	437 (8.7)	246 (8.3)
Hispanic	545 (8.9)	45 (9.1)	309 (8.8)	191 (8.9)
Non-Hispanic Asian	267 (4.7)	22 (4.6)	157 (5.0)	88 (4.3)
Other	103 (3.1)	18 (5.7)	43 (2.7)	42 (3.3)
Education, *n* (%)				
Less than high school	663 (12.3)	67 (15.0)	338 (11.1)	258 (13.6)
High school	767 (29.8)	87 (34.3)	415 (31.0)	265 (26.9)
Some college	890 (28.4)	97 (30.5)	486 (27.4)	307 (29.5)
College or above	678 (29.5)	48 (20.3)	398 (30.6)	232 (29.9)
BMI, kg/m^2^ (mean ± SD)	29.6 ± 6.3	30.5 ± 8.3	29.7 ± 6.1	29.3 ± 6.2
BMI Category, *n* (%)				
Normal/Under (<25)	699 (22.8)	71 (24.2)	356 (21.8)	272 (23.9)
Overweight (25–29.9)	1062 (35.6)	106 (32.9)	591 (35.0)	365 (37.3)
Obese (≥30)	1237 (41.6)	122 (42.9)	690 (43.2)	425 (38.8)
Smoking, *n* (%)				
Never	1520 (52.1)	119 (39.5)	841 (52.7)	560 (54.0)
Former	1079 (37.5)	125 (38.6)	562 (37.4)	392 (37.3)
Current	399 (10.4)	55 (21.9)	234 (9.9)	110 (8.6)
PIR (mean ± SD)	3.23 ± 1.55	2.72 ± 1.79	3.39 ± 1.50	3.10 ± 1.52
Sleep duration, h (mean ± SD)	7.78 ± 1.58	4.56 ± 0.91	7.26 ± 0.69	9.33 ± 0.95
PHQ-9 score (mean ± SD)	2.93 ± 3.95	4.50 ± 5.31	2.54 ± 3.57	3.19 ± 4.05
No. of chronic conditions (mean ± SD)	2.24 ± 1.64	2.48 ± 1.98	2.13 ± 1.62	2.36 ± 1.55
Multimorbidity (≥2), *n* (%)	1979 (64.0)	196 (62.1)	1037 (61.0)	746 (69.4)

**Table 2:** Survey-weighted multivariable logistic regression of sleep duration and multimorbidity (≥2 chronic conditions). NHANES 2017–March 2020.

Exposure	Model 1 (Crude)	*p*	Model 2 (Adjusted; Primary Analysis)	*p*
Short sleep < 6 h	1.05 (0.62–1.78)	0.855	0.92 (0.58–1.47)	0.726
Long sleep > 8 h	1.45 (1.09–1.91)	0.012	1.41 (1.08–1.85)	0.014
Normal 6–8 h (ref.)	1.00	—	1.00	—

**Table 3:** Sensitivity analyses for the association between long sleep duration (>8 h) and multimorbidity.

Sensitivity Analysis	*n*	Estimate (95% CI)	*p*
Main analysis (multimorbidity ≥ 2)	2998	1.41 (1.08–1.85)	0.014
A1: Outcome ≥ 3 chronic conditions	2998	1.30 (0.99–1.72)	0.061
A2: Outcome ≥ 4 chronic conditions	2998	1.12 (0.80–1.57)	0.488
A3: Outcome excluding arthritis	2998	1.31 (1.02–1.68)	0.034
A4: Outcome excluding borderline diabetes	2998	1.39 (1.05–1.84)	0.023
A5: Excluding extreme sleep (<4 h or >12 h)	2917	1.40 (1.07–1.84)	0.018
A6: +PIR + PHQ-9 + physical activity	2415	1.34 (0.91–1.96)	0.129
A7: Continuous outcome (beta, disease count, WLS)	2998	β = 0.185 (0.020 to 0.350)	0.030
A8: Continuous outcome (IRR, survey-weighted Poisson)	2998	IRR = 1.09 (1.01–1.17)	0.029
A9: +PHQ-9 only (Model 3 of Table 2)	2771	1.32 (0.95–1.82)	0.094
A10: +PHQ-8, sleep item removed	2771	1.31 (0.94–1.82)	0.101
A11: CVD diagnoses collapsed into one condition	2998	1.42 (1.09–1.86)	0.012
A12: +osteoporosis and depression, 13 conditions	2998	1.36 (1.08–1.70)	0.010
A13: +Fried-style frailty phenotype	2732	1.38 (1.00–1.90)	0.049
A14: +sedative or anxiolytic medication use	2998	1.37 (1.05–1.79)	0.021
A15: +deficit index excluding outcome-downstream items	2986	1.38 (1.03–1.85)	0.031
A16: +all four sleep-quality indicators	2565	1.37 (0.99–1.89)	0.060
A17: Excluding cancer, COPD and heart-failure cases	1914	1.31 (0.96–1.78)	0.083
A18: Multiple imputation, m = 20	3422	1.39 (1.08–1.79)	0.012
A19: Excluding refused/don’t-know on any condition item	2921	1.40 (1.06–1.85)	0.021
A20: OVER-ADJUSTED, +full mFI-11 deficit index	2998	1.18 (0.84–1.65)	0.326
A21: OVER-ADJUSTED, +number of prescription medications	2998	1.19 (0.84–1.68)	0.318
E-value, corrected for a common outcome (point estimate/lower CI)	—	1.51/1.20	
Adjusted risk difference, long sleep vs. 6–8 h	2998	7.0 percentage points (1.7 to 12.2)	

## Data Availability

All NHANES data analyzed in this study are publicly available at https://www.cdc.gov/nchs/nhanes/ (accessed on 2 June 2026). The complete analytic code is deposited as Supplementary Material rather than being available on request. It comprises the Python analysis script, including the one-hour categorical analysis, the restricted cubic spline and delete-one-PSU jackknife variance routines, the frailty and sleep-quality derivations, the g-computation for the absolute-scale contrast, and the multiple-imputation procedure; the R script used for the independent survey::svyglm cross-validation; the figure-generation script; and the machine-readable results file. The code reads the public NHANES files directly and no individual-level data are redistributed. A completed STROBE checklist and Tables S1–S18 are provided as Supplementary Material.

## Supplementary Materials

The following supporting information can be downloaded at https://www.mdpi.com/article/10.3390/healthcare14213580/s1, Table S1. Weighted prevalence of each chronic condition in the analytic sample (*n* = 2998); Table S2. Reverse-causation sensitivity analyses; Table S3. Prevalence of sleep-quality indicators by sleep-duration category; Table S4. Long sleep and multimorbidity after adjustment for sleep quality; Table S5. Joint exposure: sleep duration and sleep quality; Table S6. Frailty measures; Table S7. Long sleep and multimorbidity under different frailty adjustments; Table S8. Sedative or anxiolytic medication use and polypharmacy; Table S9. Alternative multimorbidity outcome definitions; Table S10. Missing data; Table S11. Multiple imputation versus complete-case analysis; Table S12. E-value recomputed for a common outcome; Table S13. Restricted cubic spline knot sensitivity; Table S14. Independent cross-validation of the analytic pipeline; Table S15. Complete coefficient tables for all seven models; Table S16. Subgroup estimates and formal tests of interaction; Table S17. One-hour sleep bands; Table S18. Decomposition of the fully adjusted model (Table 3, row A6); Figure S1. Two causal structures consistent with the observed data; File S1. Analysis code.
