# Supplementary material for: Long Sleep Duration and Multimorbidity in Older U.S. Adults: A Dose–Response Analysis of NHANES 2017–March 2020
Source: Healthcare (Basel). 2026 Oct 22;14(21):3580. doi: 10.3390/healthcare14213580 (PMC13762853; doi:10.3390/healthcare14213580)
Supplement: Supplementary file 1 [file healthcare-14-03580-s001.zip › Supplementary Materials Figure S1 and Tables S1-S18.pdf]

Supplementary Materials

Long Sleep Duration and Multimorbidity in Older U.S. Adults:
A Dose–Response Analysis of NHANES 2017–March 2020

Jinhua Zhang, Ki-Hyun Kim, Hyungsoo Shin*, Wansuk Choi*
Manuscript ID healthcare-4433464

All estimates account for the full NHANES complex survey design (Mobile Examination Center weight WTMECPRP, masked variance pseudo-strata SDMVSTRA and pseudo-primary sampling units SDMVPSU). Variances are estimated by the delete-one-PSU jackknife, and confidence intervals and p-values use a t reference distribution with design df = 25 (49 pseudo-PSUs minus 24 pseudo-strata). Unless stated otherwise, adjusted models control for age, sex, race/ethnicity, education, body mass index and smoking status (Model 2, the primary model).

Contents

Table S1. Weighted prevalence of each chronic condition
Table S2. Reverse-causation sensitivity analyses
Table S3. Prevalence of sleep-quality indicators by sleep-duration category
Table S4. Long sleep and multimorbidity, adjusted for sleep quality
Table S5. Joint exposure: sleep duration and sleep quality
Table S6. Frailty measures: components, and how each deficit relates to the outcome
Table S7. Long sleep and multimorbidity under different frailty adjustments
Table S8. Sedative or anxiolytic medication use and polypharmacy
Table S9. Alternative multimorbidity outcome definitions
Table S10. Missing data: included versus excluded participants, and the missingness model
Table S11. Multiple imputation versus complete-case analysis
Table S12. E-value recomputed for a common outcome
Table S13. Restricted cubic spline knot sensitivity
Table S14. Independent cross-validation: Python versus R survey
Table S15. Complete coefficient tables for all seven models
Table S16. Subgroup estimates and tests of interaction
Table S17. One-hour sleep bands
Table S18. Decomposition of the fully adjusted model: adjustment versus loss of sample
Figure S1. Two causal structures consistent with the observed data

Table S1. Weighted prevalence of each chronic condition in the analytic sample (*n* = 2,998).

Condition	n (unweighted)	Weighted %
Hypertension	1827	56.9
Arthritis	1545	52.6
Cancer (any)	641	26.2
Diabetes (incl. borderline)	944	26.0
Osteoporosis (OSQ060) - not in main definition	439	17.8
COPD	451	16.3
Coronary heart disease	293	10.8
Myocardial infarction	282	8.6
Stroke	283	8.0
Depression, PHQ-9>=10 - not in main definition	234	7.1
Liver disease	214	6.7
Congestive heart failure	228	6.2
Weak/failing kidneys	210	5.7

Percentages are survey-weighted. The eleven conditions above the two italicised rows constitute the primary multimorbidity definition. Osteoporosis and depression are shown for reference only; they are not part of the primary definition but are added in the expanded definition tested in Table S9.

Table S2. Reverse-causation sensitivity analyses.

Each row re-estimates the primary model after either restricting the sample (rows beginning "Excl.") or removing conditions from the outcome definition. The long-sleep adjusted odds ratio is shown.

Analysis	n	aOR (95% CI)	p	MM>=2 weighted %	Note
Main analysis (11 conditions, full sample)	2998	1.41 (1.08-1.85)	0.0139	64.0	reference
Excl. participants with cancer	2357	1.38 (1.06-1.80)	0.0177	56.1	
Excl. participants with COPD	2547	1.44 (1.04-1.98)	0.0282	58.5	
Excl. participants with heart failure	2770	1.40 (1.06-1.84)	0.0184	61.8	
Excl. participants with cancer/COPD/CHF	1914	1.31 (0.96-1.78)	0.0827	48.4	
Excl. participants with cancer/COPD/CHF/stroke/arthritis	948	1.47 (0.86-2.52)	0.1542	21.2	
Outcome excl. cancer, COPD, CHF (8 conditions)	2998	1.24 (0.94-1.63)	0.1235	53.7	
Outcome excl. arthritis (10 conditions)	2998	1.31 (1.02-1.68)	0.0336	48.1	
Excl. users of a sedative or anxiolytic medication	2709	1.30 (0.97-1.74)	0.0791	62.5	
Excl. clinically depressed (PHQ-9>=10)	2537	1.33 (0.96-1.85)	0.0827	62.3	
Excl. sleep-apnea symptoms (snort/stop breathing)	2607	1.40 (1.07-1.83)	0.0167	62.9	
Excl. self-rated health fair/poor	2100	1.29 (0.94-1.77)	0.1105	58.6	
Excl. hospitalised in past year	2510	1.31 (1.00-1.73)	0.0539	59.4	

Conditions that plausibly cause prolonged time in bed were removed individually and jointly. The association is robust to removing any single condition but attenuates to non-significance when cancer, chronic obstructive pulmonary disease and heart failure are excluded together, and when participants in the poorest health are excluded.

Table S3. Prevalence of sleep-quality indicators by sleep-duration category.

Indicator	n with data	short %	normal %	long %	overall %
Ever told a doctor about trouble sleeping (SLQ050)	2995	52.2	32.8	35.6	35.3
Feels overly sleepy in the daytime often or almost always (SLQ120 >= 3)	2991	37.0	23.2	25.2	25.0
Snorts, gasps or stops breathing frequently, 5+ nights/week (SLQ040 = 3)	2764	4.7	4.8	6.7	5.5
Snores frequently, 5+ nights/week (SLQ030 = 3)	2691	34.4	29.9	29.9	30.3
Any of the first three (composite poor sleep quality; snoring excluded)	2997	63.9	45.7	51.3	49.2

All percentages are survey-weighted and computed among participants with non-missing data for the indicator. NHANES variable names are given in parentheses.

Table S4. Long sleep and multimorbidity after adjustment for sleep quality.

Model	n	Long-sleep aOR (95% CI)	p
Model 2 (primary, no sleep-quality adjustment)	2998	1.41 (1.08-1.85)	0.0139
+ trouble sleeping (SLQ050)	2995	1.38 (1.06-1.81)	0.0206
+ daytime sleepiness (SLQ120)	2991	1.42 (1.07-1.88)	0.0174
+ apnea symptoms (SLQ040)	2764	1.39 (1.05-1.85)	0.0253
+ snoring (SLQ030)	2691	1.40 (1.04-1.87)	0.0265
+ all four sleep-quality indicators	2565	1.37 (0.99-1.89)	0.0603

Sample size falls across rows because the apnea-symptom and snoring items have more missing data than the other indicators.

Table S5. Joint exposure: sleep duration and sleep quality.

Joint exposure	n	aOR (95% CI)	p
Normal 6-8 h + poor sleep quality	722	2.44 (1.70-3.49)	0.0
Long >8 h + good sleep quality	551	1.47 (1.04-2.09)	0.0305
Long >8 h + poor sleep quality	511	3.00 (2.23-4.05)	0.0

Reference category: normal sleep duration (6–8 h) with good sleep quality (n = 914). Poor sleep quality is defined as any of: having told a doctor about trouble sleeping, frequent daytime sleepiness, or frequently snorting or stopping breathing during sleep. Adjusted for age, sex, race/ethnicity, education, body mass index and smoking status.

Table S6. Frailty measures.

(a) Components of the deficit-accumulation index (mFI-11), and how strongly each deficit is itself associated with the outcome

Deficit	n with data	weighted % present	OR vs multimorbidity	Causal position
Fair or poor self-rated health	2997	21.2	3.76 (2.60-5.44)	downstream of outcome
Hospitalized in the past year	2998	16.5	4.34 (2.98-6.31)	downstream of outcome
Four or more health-care visits in the past year	2929	47.4	3.62 (2.73-4.80)	downstream of outcome
Saw a mental-health professional in the past year	2998	7.2	2.49 (1.24-4.99)	downstream of outcome
Five or more prescription medications	2998	38.2	7.06 (5.03-9.90)	downstream of outcome
Sedative or anxiolytic medication use	2998	11.6	1.83 (1.17-2.87)	downstream of outcome
Depressive symptoms (PHQ-9 >= 10)	2771	7.1	3.34 (1.68-6.65)	plausibly upstream
No moderate or vigorous recreational activity	2996	54.8	1.26 (0.95-1.66)	plausibly upstream
Underweight (BMI < 18.5)	2998	0.4	0.95 (0.23-3.82)	plausibly upstream
Unintentional weight loss >= 5%	2939	9.5	1.89 (1.30-2.74)	plausibly upstream
Osteoporosis	2982	17.8	1.70 (1.24-2.33)	downstream of outcome

The final column is the reason the deficit index is NOT used as the primary frailty adjustment. Seven of the eleven deficits are health-care utilisation, medication burden, global self-rated health or another chronic disease, and each is strongly associated with the outcome (odds ratios 1.70 to 7.06). These are plausibly consequences of having accumulated chronic diagnoses rather than causes of it, so conditioning on them removes part of the outcome itself. The index correlates 0.47 with the chronic-condition count.

(b) The primary frailty measure: a partial Fried phenotype

Fried defines frailty by five criteria. This NHANES cycle supports three of them: unintentional weight loss of at least 5% in the past year, absence of moderate or vigorous recreational activity, and exhaustion (feeling tired or having little energy on more than half of days). Grip strength and gait speed were not collected in the 2017-March 2020 release. None of the three components is health-care utilisation or medication, and the resulting score correlates 0.22 with the chronic-condition count, less than half the correlation of the deficit index. It is scored 0 to 3 and was computable for 2,732 participants (91.1%); the weighted distribution was 39.4%, 43.7%, 14.2% and 2.6% for 0, 1, 2 and 3 components respectively.

Table S7. Long sleep and multimorbidity under different frailty adjustments.

Model	n	Same-sample baseline aOR	Long-sleep aOR (95% CI)	p
Model 2 (primary, no frailty adjustment)	2998	1.41 (1.08-1.85)	1.41 (1.08-1.85)	0.0139
PRIMARY: + frailty phenotype, weight loss + low activity	2937	1.45 (1.11-1.90)	1.43 (1.08-1.89)	0.0135
+ frailty phenotype including exhaustion (0-3 components)	2732	1.43 (1.06-1.94)	1.38 (1.00-1.90)	0.049
+ exhaustion criterion alone	2781	1.41 (1.04-1.91)	1.36 (0.98-1.89)	0.0612
+ frailty phenotype + PHQ-7 (sleep and energy items removed)	2722	1.43 (1.06-1.93)	1.33 (0.95-1.85)	0.09
+ deficit index, downstream items removed (FI-4)	2986	1.42 (1.08-1.87)	1.38 (1.03-1.85)	0.0306
OVER-ADJUSTED: + full mFI-11 (contains outcome-downstream items)	2998	1.41 (1.08-1.85)	1.18 (0.84-1.65)	0.3257
OVER-ADJUSTED: + the five downstream items only (FI-5)	2998	1.41 (1.08-1.85)	1.19 (0.85-1.67)	0.2881
Restricted to non-frail on the phenotype (<2 components)	2205	1.42 (1.03-1.97)	1.42 (1.03-1.97)	0.0337

The association is essentially unchanged after adjustment for the Fried-style phenotype (1.38, 95% CI 1.00 to 1.90) and after adjustment for a deficit index from which the outcome-downstream items have been removed (1.38, 1.03 to 1.85). It is also unchanged, and remains significant, when the analysis is restricted to participants who are non-frail on the phenotype (1.42, 1.03 to 1.97) - which is the opposite of what would be expected if frailty were generating the association. The two rows labelled OVER-ADJUSTED are reported for transparency and should not be read as evidence against the association: conditioning on the full deficit index, or on the five downstream deficits alone, removes variation that is itself a consequence of the outcome, and both give essentially the same attenuated estimate (1.18 and 1.19).

Table S8. Sedative or anxiolytic medication use and polypharmacy.

(a) Distribution by sleep-duration category

Sleep category	sedative or anxiolytic %	polypharmacy (>=5) %	mean n prescriptions
short	12.6	42.7	4.22
normal	9.3	32.7	3.69
long	15.2	46.0	4.56

(b) Effect of adjustment on the long-sleep association

Model	n	Long-sleep aOR (95% CI)	p
Model 2 (primary)	2998	1.41 (1.08-1.85)	0.0139
+ sedative or anxiolytic medication use	2998	1.37 (1.05-1.79)	0.0209
+ number of prescription medications	2998	1.19 (0.84-1.68)	0.3181
OVER-ADJUSTED: + sedative + n medications + full mFI-11	2998	1.17 (0.81-1.68)	0.3919

Medication use was derived from the NHANES prescription-medication file (P_RXQ_RX). The exposure is deliberately named sedative or anxiolytic medication rather than sedative-hypnotic: it comprises benzodiazepine-receptor agonists, benzodiazepines, sedating antidepressants and antipsychotics, melatonin-receptor and orexin-receptor agonists, sedating antihistamines and barbiturates, and of the 347 prescriptions it flags in this sample only 18% are agents licensed specifically as hypnotics. The class is dominated by trazodone, alprazolam, lorazepam and clonazepam, which are prescribed for a range of indications, so the variable should not be read as a measure of pharmacological treatment for insomnia.

Table S9. Alternative multimorbidity outcome definitions.

Outcome definition	n conditions	n	MM>=2 weighted %	aOR (95% CI)	p
Main definition (primary)	11	2998	64.0	1.41 (1.08-1.85)	0.0139
CVD collapsed: CHF+CHD+MI -> one condition	9	2998	63.9	1.42 (1.09-1.86)	0.0124
+ osteoporosis	12	2998	67.2	1.36 (1.08-1.71)	0.012
+ osteoporosis + depression (PHQ-9>=10)	13	2998	67.6	1.36 (1.08-1.70)	0.0098
CVD collapsed + osteoporosis + depression	11	2998	67.4	1.37 (1.09-1.71)	0.0085
Excluding arthritis	10	2998	48.1	1.31 (1.02-1.68)	0.0336
Strict (non-borderline) diabetes	11	2998	63.2	1.39 (1.05-1.84)	0.0233

The collapsed-cardiovascular definition combines congestive heart failure, coronary heart disease and myocardial infarction into a single indicator, addressing the possibility that a single cardiovascular trajectory inflates the condition count. Depression is defined as a PHQ-9 score of 10 or above.

Among the 521 participants reporting at least one of the three cardiovascular diagnoses, 302 reported one, 156 reported two and 63 reported all three.

Table S10. Missing data.

(a) Included versus excluded participants aged 60 years and over

Variable	Included mean	n incl	Excluded mean	n excl	p (Welch)
Age, years	69.64	2998	72.86	424	0.0
Female, %	0.49	2998	0.52	424	0.1915
PIR	2.63	2587	2.46	297	0.0739
Sleep duration, h	7.75	2998	8.08	384	0.0012
N chronic conditions	2.31	2998	2.62	424	0.0004
Multimorbidity >=2, %	0.66	2998	0.73	424	0.0033
PHQ-9	3.05	2771	6.12	89	0.0
BMI	29.73	2998	29.61	43	0.9243

(b) Survey-weighted logistic model for the probability of exclusion

Predictor	OR	Lower 95% CI	Upper 95% CI	p
RIDAGEYR	1.1034	1.0457	1.1643	0.0009
fem	0.958	0.5662	1.621	0.8679
ncond	1.0734	0.9373	1.2292	0.2922

Little's test of the missing-completely-at-random assumption, applied to sleep duration, body mass index, poverty income ratio, PHQ-9 score, age and condition count, gave chi-square = 267.6 on 58 degrees of freedom across 16 missingness patterns ($p < 0.001$). The MCAR assumption is therefore rejected. Older age predicted exclusion, consistent with data missing at random conditional on observed covariates, which is the assumption under which the multiple imputation in Table S11 is valid.

Table S11. Multiple imputation versus complete-case analysis.

Approach	n	aOR (95% CI)	p	aOR
Complete case (primary)	2998	1.41 (1.08-1.85)	0.0139	
Multiple imputation (m=20, Rubin)	3422	1.39 (1.08-1.79)	0.0118	
R mice + survey, m = 20 (Rubin pooling)	3422		0.0317	1.40 (1.04-1.87)

Twenty imputed datasets were generated by chained equations and combined by Rubin's rules, independently in Python and in R (mice). Imputation was performed in all 3,422 participants aged 60 and over. The imputed estimate is close to the complete-case estimate, indicating that the exclusion of 424 participants did not materially bias the primary result.

Table S12. E-value recomputed for a common outcome.

Estimate	OR	Baseline risk p0 (6-8 h stratum)	RR (VanderWeele-Ding conversion)	E-value (converted RR)	RR (sqrt-OR approximation)	E-value (sqrt-OR)	E-value if OR treated as RR
Point estimate	1.414	0.61	1.129	1.51	1.189	1.66	2.18
Lower confidence limit	1.08	0.61	1.03	1.2	1.039	1.24	1.37

Multimorbidity affects 64.0% of the analytic sample, so the odds ratio substantially overstates the risk ratio and an E-value computed directly from the odds ratio is too large. Applying the VanderWeele and Ding conversion, with a baseline risk of 0.610 in the 6–8 h reference stratum, gives a corrected E-value of 1.51 for the point estimate and 1.20 for the lower confidence limit. The E-values computed directly on the odds-ratio scale would be 2.18 and 1.37; the corrected values are reported in the manuscript and interpreted as a weak bound.

Table S13. Restricted cubic spline knot sensitivity.

Specification	Knots (hours)	aOR at 8 h	aOR at 9 h	aOR at 10 h	P-nonlinearity
4 knots, Harrell canonical (5, 35, 65, 95)	5.0 / 7.0 / 8.5 / 10.5	1.18 (0.97-1.43)	1.46 (1.08-1.97)	1.76 (1.28-2.43)	0.0048
4 knots, symmetric placement (5, 27.5, 72.5, 95)	5.0 / 7.0 / 9.0 / 10.5	1.17 (0.98-1.41)	1.48 (1.08-2.02)	1.78 (1.28-2.47)	0.0051
3 knots, Harrell canonical (10, 50, 90)	6.0 / 8.0 / 10.0	1.05 (0.94-1.18)	1.27 (1.05-1.54)	1.65 (1.20-2.28)	0.0377
5 knots, Harrell canonical (5, 27.5, 50, 72.5, 95)	5.0 / 7.0 / 8.0 / 9.0 / 10.5	1.12 (0.85-1.47)	1.50 (1.08-2.09)	1.81 (1.27-2.58)	0.0115

The primary specification in the manuscript is Harrell's canonical four-knot placement at the 5th, 35th, 65th and 95th percentiles. The symmetric placement at the 5th, 27.5th, 72.5th and 95th percentiles is retained in the second row for comparison. The J-shape and its statistical significance are preserved across all four specifications.

Table S14. Independent cross-validation of the analytic pipeline.

Estimate	Implementation	aOR (95% CI)	p
Model 1 (crude), long sleep >8 h	Python / statsmodels, delete-one-PSU jackknife (the primary analysis pipeline)	1.448 (1.095-1.915)	0.0116
Model 1 (crude), long sleep >8 h	R survey::svyglm, JKn jackknife replicates	1.448 (1.095-1.915)	0.0116
Model 1 (crude), long sleep >8 h	R survey::svyglm, Taylor linearisation	1.448 (1.095-1.914)	0.0115
Model 1 (crude), short sleep <6 h	Python / statsmodels, delete-one-PSU jackknife (the primary analysis pipeline)	1.049 (0.618-1.778)	0.8548
Model 1 (crude), short sleep <6 h	R survey::svyglm, JKn jackknife replicates	1.049 (0.618-1.778)	0.8548
Model 1 (crude), short sleep <6 h	R survey::svyglm, Taylor linearisation	1.049 (0.621-1.772)	0.8538
Model 2 (primary, adjusted), long sleep >8 h	Python / statsmodels, delete-one-PSU jackknife (the primary analysis pipeline)	1.414 (1.080-1.852)	0.0139
Model 2 (primary, adjusted), long sleep >8 h	R survey::svyglm, JKn jackknife replicates	1.414 (1.080-1.852)	0.0139
Model 2 (primary, adjusted), long sleep >8 h	R survey::svyglm, Taylor linearisation	1.414 (1.081-1.850)	0.0136
Model 2 (primary, adjusted), short sleep <6 h	Python / statsmodels, delete-one-PSU jackknife (the primary analysis pipeline)	0.923 (0.579-1.471)	0.7263
Model 2 (primary, adjusted), short sleep <6 h	R survey::svyglm, JKn jackknife replicates	0.923 (0.579-1.471)	0.7263
Model 2 (primary, adjusted), short sleep <6 h	R survey::svyglm, Taylor linearisation	0.923 (0.581-1.467)	0.7247
Model 3 (+ PHQ-9), long sleep >8 h	Python / statsmodels, delete-one-PSU jackknife (the primary analysis pipeline)	1.317 (0.951-1.823)	0.0940
Model 3 (+ PHQ-9), long sleep >8 h	R survey::svyglm, JKn jackknife replicates	1.317 (0.951-1.823)	0.0940
Model 3 (+ PHQ-9), long sleep >8 h	R survey::svyglm, Taylor linearisation	1.317 (0.952-1.821)	0.0930
Model 3 (+ PHQ-9), short sleep <6 h	Python / statsmodels, delete-one-PSU jackknife (the primary analysis pipeline)	0.783 (0.491-1.250)	0.2914
Model 3 (+ PHQ-9), short sleep <6 h	R survey::svyglm, JKn jackknife replicates	0.783 (0.491-1.250)	0.2914
Model 3 (+ PHQ-9), short sleep <6 h	R survey::svyglm, Taylor linearisation	0.783 (0.492-1.246)	0.2887
Model 3 (+ PHQ-9), PHQ-9 per point	Python / statsmodels, delete-one-PSU jackknife (the primary analysis pipeline)	1.114 (1.074-1.156)	0.0000
Model 3 (+ PHQ-9), PHQ-9 per point	R survey::svyglm, JKn jackknife replicates	1.114 (1.074-1.156)	0.0000
Model 3 (+ PHQ-9), PHQ-9 per point	R survey::svyglm, Taylor linearisation	1.114 (1.074-1.156)	0.0000
RCS spline aOR at 9 h vs 7 h	R survey::svyglm, Taylor linearisation	1.46	P-nonlinearity 0.0195
RCS spline aOR at 9 h vs 7 h	Python, delete-one-PSU jackknife	1.46 (1.08-1.97)	P-nonlinearity 0.0048
Multiple imputation (m = 20), long sleep	Python, chained equations + Rubin pooling	1.395 (1.084-1.794)	0.0118
Multiple imputation (m = 20), long sleep	R mice + survey, Rubin pooling	1.40 (1.04-1.87)	0.0317

Every estimate in Table 2 of the manuscript, that is the long-sleep and short-sleep odds ratios from all three models together with the PHQ-9 per-point coefficient, was refitted in R using the survey package under two variance estimators: Taylor-series linearisation (the default recommended by NCHS) and a JKn delete-one-PSU jackknife replicate-weight design. R independently returns design df = 25. All point estimates agree to three decimal places, and all confidence limits agree to within 0.006, the Python and R jackknife designs agreeing exactly and the Taylor-linearisation limits differing only in the third decimal place. Two quantities do differ materially between implementations, and we report both rather than choosing the more favourable one. First, the test for non-linearity of the spline gives p = 0.005 by jackknife and p = 0.020 by Taylor linearisation; both reject linearity. Second, the multiple-imputation estimate is 1.395 (1.084 to 1.794), p = 0.012 with the Python imputation engine and 1.40 (1.04 to 1.87), p = 0.032 with R mice; the point estimates are effectively identical and the difference lies in the width of the interval, which reflects the two packages' different degrees-of-freedom corrections. Complete analytic code in Python (revision_analyses.py, make_figures.py, build_crossvalidation_table.py and a Colab notebook) and in R (crossvalidate_R.R) is deposited with this article.

Table S15. Complete coefficient tables for all seven models.

Model 1 (crude)

Term	aOR (95% CI)	p	n
Short sleep <6 h (vs 6-8 h)	1.05 (0.62-1.78)	0.8548	2998
Long sleep >8 h (vs 6-8 h)	1.45 (1.09-1.91)	0.0116	2998

Model 2 (primary, adjusted)

Term	aOR (95% CI)	p	n
Short sleep <6 h (vs 6-8 h)	0.92 (0.58-1.47)	0.7263	2998
Long sleep >8 h (vs 6-8 h)	1.41 (1.08-1.85)	0.0139	2998
Age (per year)	1.07 (1.05-1.09)	<0.001	2998
Female (vs male)	0.95 (0.76-1.18)	0.6017	2998
Hispanic (vs non-Hispanic White)	0.89 (0.58-1.36)	0.5694	2998
Non-Hispanic Black (vs non-Hispanic White)	1.14 (0.85-1.54)	0.3736	2998
Non-Hispanic Asian (vs non-Hispanic White)	1.10 (0.71-1.69)	0.6645	2998
Other or multiracial (vs non-Hispanic White)	3.42 (1.73-6.74)	0.001	2998
<9th grade (vs college graduate)	1.40 (0.89-2.21)	0.1358	2998
9-11th grade (vs college graduate)	1.45 (0.94-2.23)	0.0904	2998
High school/GED (vs college graduate)	1.70 (1.22-2.37)	0.003	2998
Some college (vs college graduate)	1.53 (1.10-2.12)	0.0127	2998
Obese >=30 (vs <25)	3.24 (2.10-4.98)	<0.001	2998
Overweight 25-29.9 (vs <25)	1.54 (1.07-2.20)	0.0207	2998
Current smoker (vs never)	1.91 (1.33-2.75)	0.0012	2998
Former smoker (vs never)	1.68 (1.27-2.23)	<0.001	2998

Model 3 (+ PHQ-9)

Term	aOR (95% CI)	p	n
Short sleep <6 h (vs 6-8 h)	0.78 (0.49-1.25)	0.2914	2771
Long sleep >8 h (vs 6-8 h)	1.32 (0.95-1.82)	0.094	2771
PHQ-9 score (per point)	1.11 (1.07-1.16)	<0.001	2771
Age (per year)	1.07 (1.05-1.09)	<0.001	2771
Female (vs male)	0.84 (0.66-1.06)	0.1387	2771
Hispanic (vs non-Hispanic White)	0.79 (0.51-1.24)	0.2938	2771
Non-Hispanic Black (vs non-Hispanic White)	1.15 (0.86-1.54)	0.3164	2771
Non-Hispanic Asian (vs non-Hispanic White)	1.29 (0.78-2.12)	0.3067	2771
Other or multiracial (vs non-Hispanic White)	3.00 (1.55-5.83)	0.0022	2771
<9th grade (vs college graduate)	1.44 (0.89-2.33)	0.1355	2771
9-11th grade (vs college graduate)	1.52 (0.90-2.54)	0.1095	2771
High school/GED (vs college graduate)	1.57 (1.10-2.24)	0.015	2771
Some college (vs college graduate)	1.46 (1.03-2.08)	0.0357	2771
Obese >=30 (vs <25)	3.16 (2.01-4.97)	<0.001	2771
Overweight 25-29.9 (vs <25)	1.63 (1.10-2.42)	0.0164	2771
Current smoker (vs never)	1.60 (1.06-2.41)	0.0274	2771
Former smoker (vs never)	1.58 (1.20-2.08)	0.0022	2771

Model 3b (+ PHQ-8, sleep item removed)

Term	aOR (95% CI)	p	n
Short sleep <6 h (vs 6-8 h)	0.80 (0.50-1.30)	0.3574	2771
Long sleep >8 h (vs 6-8 h)	1.31 (0.94-1.82)	0.1009	2771
PHQ-8 score (per point)	1.13 (1.08-1.19)	<0.001	2771
Age (per year)	1.07 (1.05-1.09)	<0.001	2771
Female (vs male)	0.85 (0.67-1.09)	0.1911	2771

Hispanic (vs non-Hispanic White)	0.79 (0.50-1.25)	0.3033	2771
Non-Hispanic Black (vs non-Hispanic White)	1.15 (0.86-1.52)	0.3304	2771
Non-Hispanic Asian (vs non-Hispanic White)	1.29 (0.79-2.12)	0.2982	2771
Other or multiracial (vs non-Hispanic White)	3.08 (1.59-5.99)	0.0018	2771
<9th grade (vs college graduate)	1.44 (0.88-2.35)	0.1372	2771
9-11th grade (vs college graduate)	1.50 (0.91-2.50)	0.1095	2771
High school/GED (vs college graduate)	1.58 (1.10-2.26)	0.0151	2771
Some college (vs college graduate)	1.46 (1.02-2.09)	0.0376	2771
Obese >=30 (vs <25)	3.18 (2.02-4.98)	<0.001	2771
Overweight 25-29.9 (vs <25)	1.62 (1.10-2.39)	0.0163	2771
Current smoker (vs never)	1.63 (1.07-2.47)	0.0233	2771
Former smoker (vs never)	1.60 (1.21-2.10)	0.0018	2771

Model 4 (+ PIR + PHQ-9 + vigorous activity)

Term	aOR (95% CI)	p	n
Short sleep <6 h (vs 6-8 h)	0.80 (0.52-1.21)	0.2714	2415
Long sleep >8 h (vs 6-8 h)	1.34 (0.91-1.96)	0.129	2415
Poverty income ratio (per unit)	0.95 (0.86-1.06)	0.3433	2415
PHQ-9 score (per point)	1.12 (1.07-1.16)	<0.001	2415
Vigorous recreational activity (yes vs no)	1.24 (0.83-1.86)	0.2714	2415
Age (per year)	1.06 (1.04-1.09)	<0.001	2415
Female (vs male)	0.86 (0.68-1.08)	0.1823	2415
Hispanic (vs non-Hispanic White)	0.65 (0.38-1.12)	0.1135	2415
Non-Hispanic Black (vs non-Hispanic White)	1.07 (0.78-1.47)	0.649	2415
Non-Hispanic Asian (vs non-Hispanic White)	1.43 (0.84-2.43)	0.1768	2415
Other or multiracial (vs non-Hispanic White)	2.13 (1.20-3.79)	0.012	2415
<9th grade (vs college graduate)	1.58 (0.88-2.86)	0.1231	2415
9-11th grade (vs college graduate)	1.58 (0.91-2.76)	0.1006	2415
High school/GED (vs college graduate)	1.64 (1.14-2.37)	0.0095	2415
Some college (vs college graduate)	1.56 (1.12-2.17)	0.0101	2415
Obese >=30 (vs <25)	3.40 (2.11-5.50)	<0.001	2415
Overweight 25-29.9 (vs <25)	1.67 (1.11-2.53)	0.0168	2415
Current smoker (vs never)	1.52 (0.92-2.53)	0.0988	2415
Former smoker (vs never)	1.71 (1.31-2.23)	<0.001	2415

Model 5 (+ frailty index) [OVER-ADJUSTED: contains items that are consequences of the outcome; reported for transparency only]

Term	aOR (95% CI)	p	n
Short sleep <6 h (vs 6-8 h)	0.75 (0.45-1.24)	0.2459	2998
Long sleep >8 h (vs 6-8 h)	1.18 (0.84-1.65)	0.3257	2998
Deficit index mFI-11 (per 1 unit, i.e. all 11 deficits present)	418.86 (148.91-1178.16)	<0.001	2998
Age (per year)	1.04 (1.02-1.06)	<0.001	2998
Female (vs male)	0.76 (0.58-0.99)	0.0428	2998
Hispanic (vs non-Hispanic White)	0.80 (0.53-1.21)	0.2746	2998
Non-Hispanic Black (vs non-Hispanic White)	1.10 (0.82-1.48)	0.5017	2998
Non-Hispanic Asian (vs non-Hispanic White)	1.13 (0.71-1.81)	0.5864	2998
Other or multiracial (vs non-Hispanic White)	2.39 (1.37-4.15)	0.0033	2998
<9th grade (vs college graduate)	0.87 (0.47-1.61)	0.6379	2998
9-11th grade (vs college graduate)	0.93 (0.58-1.47)	0.7379	2998
High school/GED (vs college graduate)	1.44 (1.02-2.04)	0.0396	2998
Some college (vs college graduate)	1.30 (0.91-1.85)	0.1433	2998
Obese >=30 (vs <25)	2.94 (1.74-4.97)	<0.001	2998
Overweight 25-29.9 (vs <25)	1.76 (1.17-2.63)	0.0083	2998
Current smoker (vs never)	1.51 (0.94-2.42)	0.0842	2998
Former smoker (vs never)	1.61 (1.19-2.16)	0.0031	2998

Model 6 (+ sleep-quality indicators)

Term	aOR (95% CI)	p	n
Short sleep <6 h (vs 6-8 h)	0.71 (0.43-1.17)	0.1681	2565
Long sleep >8 h (vs 6-8 h)	1.37 (0.99-1.89)	0.0603	2565
Told a doctor about trouble sleeping	1.74 (1.18-2.56)	0.0067	2565
Daytime sleepiness (per level)	1.37 (1.22-1.54)	<0.001	2565
Snorts/stops breathing frequently	1.29 (0.59-2.81)	0.513	2565
Snores frequently	0.94 (0.64-1.36)	0.7156	2565
Age (per year)	1.07 (1.05-1.10)	<0.001	2565
Female (vs male)	0.88 (0.71-1.10)	0.2462	2565
Hispanic (vs non-Hispanic White)	0.98 (0.64-1.51)	0.9392	2565
Non-Hispanic Black (vs non-Hispanic White)	1.16 (0.85-1.59)	0.3268	2565
Non-Hispanic Asian (vs non-Hispanic White)	1.27 (0.77-2.09)	0.3364	2565
Other or multiracial (vs non-Hispanic White)	2.59 (1.37-4.89)	0.0049	2565
<9th grade (vs college graduate)	1.36 (0.81-2.31)	0.2343	2565
9-11th grade (vs college graduate)	1.39 (0.77-2.50)	0.2624	2565
High school/GED (vs college graduate)	1.74 (1.17-2.58)	0.008	2565
Some college (vs college graduate)	1.51 (1.02-2.24)	0.0385	2565
Obese >=30 (vs <25)	3.07 (1.85-5.09)	<0.001	2565
Overweight 25-29.9 (vs <25)	1.55 (1.00-2.40)	0.0497	2565
Current smoker (vs never)	1.81 (1.25-2.62)	0.0029	2565
Former smoker (vs never)	1.63 (1.21-2.19)	0.0025	2565

All odds ratios from every model fitted in the manuscript are reported here in full, as requested. Model 2 is the primary model.

Table S16. Subgroup estimates and formal tests of interaction.

Subgroup	n	aOR (95% CI)	p	P-interaction
Overall	2998	1.41 (1.08–1.85)	0.0139	
Male	1530	1.63 (1.18–2.23)	0.0041	0.177
Female	1468	1.29 (0.89–1.86)	0.1709	
Age 60-69	1597	1.34 (0.89–2.01)	0.1525	0.550
Age >=70	1401	1.57 (1.11–2.23)	0.0129	
BMI <25	699	0.84 (0.52–1.35)	0.4594	0.027
BMI 25-29.9	1062	1.62 (0.94–2.79)	0.0775	
BMI >=30	1237	1.78 (1.21–2.62)	0.0051	
Never smoker	1520	1.28 (0.91–1.80)	0.1443	0.436
Former smoker	1079	1.59 (0.88–2.89)	0.1213	
Current smoker	399	1.97 (0.96–4.06)	0.0648	
PIR <1.85	1077	1.09 (0.75–1.57)	0.6404	0.115
PIR >=1.85	1510	1.49 (1.01–2.20)	0.0431	

Interaction tests are computed on the same categorisation that is displayed in Figure 3, so that the test and the strata answer the same question. Smoking status uses a two-degree-of-freedom joint test. Four of the five tests are null; the body mass index test reaches P = 0.027, which does not survive correction for multiplicity across the five tests (Bonferroni 0.137) and is reported as hypothesis-generating.

Table S17. One-hour sleep bands.

Sleep band	n	aOR (95% CI)	p
<6 h	299	0.96 (0.58-1.58)	0.865
6-7 h	400	1.16 (0.68-1.97)	0.5816
7-8 h (reference)	1237	1.00 (reference)	
>8 to <9 h	229	1.08 (0.67-1.73)	0.7505
>=9 h	833	1.65 (1.22-2.25)	0.0025

Reference band 7–8 h inclusive. This finer categorisation shows that the elevation in odds is confined to the ≥9 h band; the band immediately above the conventional 8 h cut-point shows no elevation.

Table S18. Decomposition of the fully adjusted model (Table 3, row A6).

Specification	n	aOR (95% CI)	p
Model 2, full sample	2998	1.41 (1.08-1.85)	0.0139
Model 2, restricted to the S6 complete cases, no new covariates	2415	1.42 (1.00-2.02)	0.0494
S6, same complete cases, with PIR + PHQ-9 + physical activity	2415	1.34 (0.91-1.96)	0.129

The fully adjusted model adds the poverty income ratio, the PHQ-9 score and physical activity, and in doing so loses 583 participants to item non-response. Refitting the primary model on that same complete-case subsample, without the three additional covariates, isolates the two contributions: the point estimate is essentially unchanged (1.41 to 1.42) and the confidence interval widens purely through the loss of sample, after which the three covariates move the estimate only from 1.42 to 1.34. Most of the attenuation observed for this model therefore reflects reduced precision rather than confounding control.

Figure S1. Two causal structures consistent with the observed data.

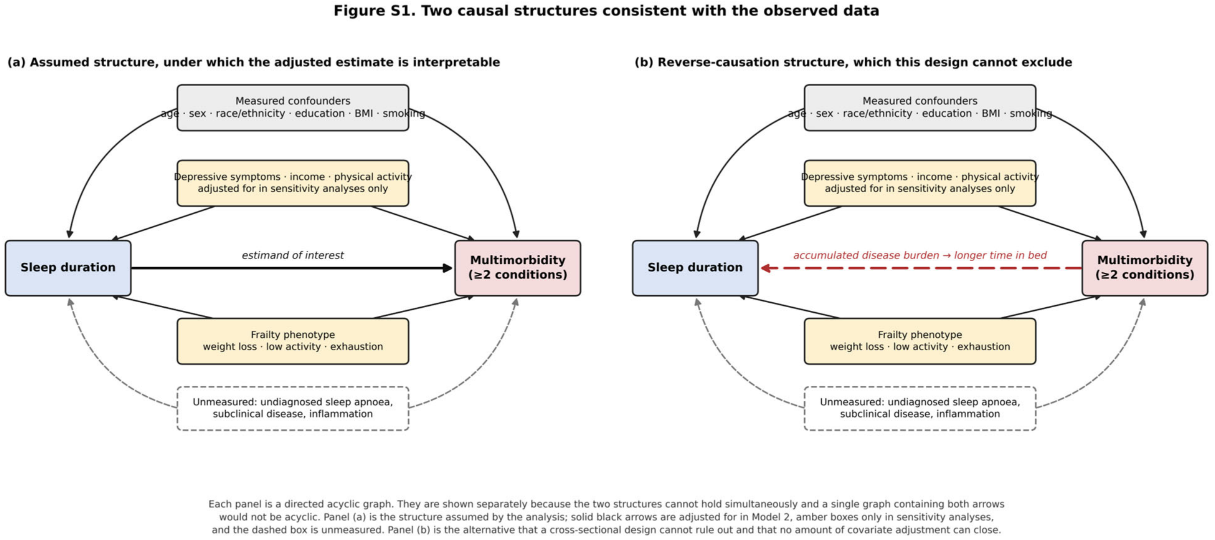

Each panel is a directed acyclic graph. The two are shown separately because the structures cannot hold simultaneously and a single graph containing both arrows would not be acyclic. Panel (a) is the structure assumed by the analysis: solid boxes are measured variables, the dashed box is unmeasured, and amber boxes are adjusted for only in sensitivity analyses because a single-visit design cannot classify them as confounders or mediators. Panel (b) is the reverse-causation structure, which no covariate adjustment can close and which the E-value does not bound.
